# Multi-omics integration of methyltransferase-like protein family reveals clinical outcomes and functional signatures in human cancer

**DOI:** 10.1038/s41598-021-94019-5

**Published:** 2021-07-20

**Authors:** Ion John Campeanu, Yuanyuan Jiang, Lanxin Liu, Maksymilian Pilecki, Alvina Najor, Era Cobani, Morenci Manning, Xiaohong Mary Zhang, Zeng-Quan Yang

**Affiliations:** 1 Department of Oncology, Wayne State University School of Medicine, Detroit, MI USA; 2 Molecular Therapeutics Program, Barbara Ann Karmanos Cancer Institute, 4100 John R Street, HWCRC 815, Detroit, MI 48201 USA

**Keywords:** Cancer genomics, Methylation, Molecular medicine

## Abstract

Human methyltransferase-like (METTL) proteins transfer methyl groups to nucleic acids, proteins, lipids, and other small molecules, subsequently playing important roles in various cellular processes. In this study, we performed integrated genomic, transcriptomic, proteomic, and clinicopathological analyses of 34 METTLs in a large cohort of primary tumor and cell line data. We identified a subset of METTL genes, notably *METTL1, METTL7B*, and *NTMT1*, with high frequencies of genomic amplification and/or up-regulation at both the mRNA and protein levels in a spectrum of human cancers. Higher METTL1 expression was associated with high-grade tumors and poor disease prognosis. Loss-of-function analysis in tumor cell lines indicated the biological importance of METTL1, an m^7^G methyltransferase, in cancer cell growth and survival. Furthermore, functional annotation and pathway analysis of METTL1-associated proteins revealed that, in addition to the METTL1 cofactor WDR4, RNA regulators and DNA packaging complexes may be functionally interconnected with METTL1 in human cancer. Finally, we generated a crystal structure model of the METTL1–WDR4 heterodimeric complex that might aid in understanding the key functional residues. Our results provide new information for further functional study of some METTL alterations in human cancer and might lead to the development of small inhibitors that target cancer-promoting METTLs.

## Introduction

Human methyltransferase-like (METTL) proteins belong to a superfamily of S-adenosyl methionine (SAM)-dependent enzymes that transfer methyl groups to nucleic acids, proteins, lipids, and small molecules^1^. Based on conserved amino acid sequences and crystal structures of their catalytic domains, methyltransferases are divided into at least five families^1^. More than 100 methyltransferases have a seven-beta-strand (Rossmann) fold catalytic domain and are classified as class I enzymes. In addition to the METTL subfamily, class I methyltransferases also contain the tRNA methyltransferase (TRMT), NOP2/Sun RNA methyltransferase (NSUN), and protein arginine methyltransferase (PRMT) subfamilies. Class I methyltransferases were previously shown to be essential in epigenetic control, gene expression, lipid biosynthesis, protein stability, RNA metabolism, and cell differentiation^1,2^. Thus, class I methyltransferases, including METTLs, regulate a variety of cellular processes and biological functions.

Human METTLs have been implicated in development and progression of various human diseases, including cancers^2,3^. METTL1 is linked to several human cancers, including hepatocellular carcinoma, with combined METTL1 and NSUN2 knockdown increasing sensitivity to 5-fluorouracil in tumor lines^4,5^. METTL3, in complex with its partner METTL14, catalyzes N6-methyladenosine (m^6^A) methylation in mRNAs and noncoding RNAs and promotes oncogene expression and cancer cell growth in leukemia and some solid tumors^6,7^. METTL13, or EEF1AKNMT (eEF1A lysine and N-terminal methyltransferase), catalyzes the methylation of eEF1A (eukaryotic elongation factor 1A), regulating translation and elongation and promoting tumorigenesis in lung and pancreatic cancer^8^. A very recent study revealed that METTL18 is a histidine methyltransferase that regulates translation in cancer cells^9^. In addition to cancer, mutations and copy number alterations in METTLs can cause multiple neurological and multisystemic disorders^10,11^. Thus, previous studies demonstrate that METTLs are important regulators of cellular processes, which, if derailed, may result in the development of a multitude of diseases and disorders, including cancer. However, despite the emerging knowledge about the roles of METTLs in disease, the expression landscape and clinical relevance of METTLs in cancer have not been investigated systematically.

**Figure 1:**
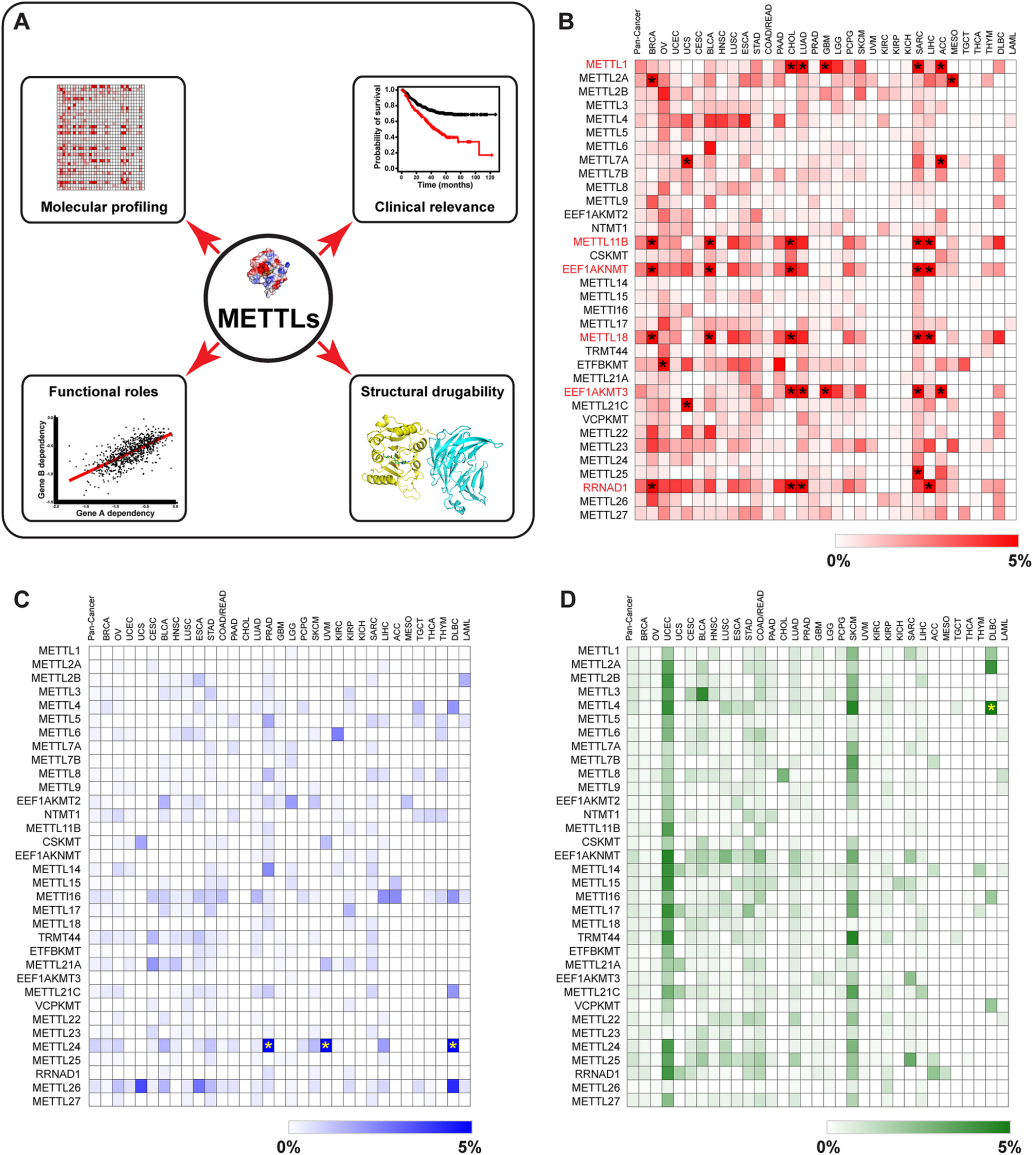
Molecular profiling of METTLs in human cancer. (**A**) Schematic of the strategy to identify potential METTLs that have important roles in cancer progression and serve as novel therapeutic targets. (**B–D**) Heatmaps showing the frequencies of METTL amplification (red), deep deletion (blue), and mutation (green) across all 32 TCGA tumor types. Heatmap was generated using Morpheus software from the Broad Institute (https://software.broadinstitute.org/morpheus/). METTL genes with amplification greater than 2% in Pan-Cancer were highlighted with red text, and individual tumors with more than 5% amplification, deletion, or mutation were marked with an asterisk.

In this study, we hypothesized that METTLs with recurrent genetic alterations may play important roles in cancer progression and can serve as novel therapeutic targets for cancer treatment (Fig. 1A). The availability of genomic, transcriptomic, and proteomic profiles across a broad range of cancers from TCGA (The Cancer Genome Atlas) and CPTAC (Clinical Proteomic Tumor Analysis Consortium) projects, as well as large-scale loss-of-function screens of cancer cell lines, provide an unprecedented opportunity to investigate genetic and proteomic alterations of METTLs associated with molecular signatures, clinical prognosis, and their functional importance in cancer in great depth. In this study, we utilized an unbiased approach to examine genetic and transcriptomic alterations of 34 METTLs in multiple in vitro and clinical datasets. We investigated METTL expression levels as they relate to clinical features such as tumor grade and survival in a large cohort of tumor samples, focusing on lung cancer. Then, we focused on a top candidate, METTL1, and investigated functional annotation and pathway enrichment of METTL1-associated proteins in human cancer. Finally, we analyzed the functional key residues of METTL1 and generated a crystal structure model of human METTL1 together with its cofactor WDR4 using molecular modeling and simulation approaches. Our results provide new information for further functional study of some METTL alterations in human cancer and might lead to the development of small inhibitors that target cancer-promoting METTLs.

## Results

### Somatic copy number alterations and mutations of METTLs across cancers

In the human genome, there are 34 encoded METTL genes with identified or putative methyltransferases targeting RNAs, proteins, lipids, or other small molecules (Supplementary Table S1, Fig. S1)^1,12^. To identify novel genomic alterations of METTLs in human cancer, we determined their somatic copy number alterations (CNA) and mutation profiles in over 10,000 TCGA tumor samples across 32 tumor types (Supplementary Table S2)^13^. The CNA and mutation data of 10,712 samples from the TCGA Pan-Cancer Atlas were obtained from cBioPortal^14^. METTL copy number was generated by the GISTIC (Genomic Identification of Significant Targets in Cancer) algorithm. The TCGA Pan-Cancer cohort showed six genes [*METTL1*, *METTL11B*, *EEF1AKNMT*, *METTL18*, *EEF1AKMT3* (*METTL21B*), *RRNAD1* (*METTL25B*)] with high-level amplifications above 2% (Fig. 1B, Supplementary Table S3). No genes showed homozygous deletions or somatic mutations above 2% in the TCGA Pan-Cancer cohort (Fig. 1C,D, Supplementary Tables S4, S5). The most mutated METTL gene is *EEF1AKNMT*, which had 129 mutations, including 111 missense, 13 truncating, 3 splice, and 2 fusion mutations.

We also performed copy number analyses of 34 METTL genes in 32 individual TCGA tumor types, finding significant variations across tumor types. Eleven METTLs had high-level amplifications above 5% in at least one tumor type (Fig. 1B, Supplementary Table S3). Among them, *METTL1* had high-level amplifications above 5% in five TCGA tumor types: lung adenocarcinoma (LUAD, 5.29%), glioblastoma (GBM, 13.57%), sarcoma (SARC, 17.00%), adrenocortical carcinoma (ACC, 6.74%), and cholangiocarcinoma (CHOL, 5.56%). *METTL2A* had high-level amplifications in two TCGA tumor types: breast cancer (BRCA, 6.73%) and mesothelioma (MESO, 5.75%). *EEF1AKNMT*, the most mutated METTL gene, had high-level amplifications above 5% in BRCA (6.64%), bladder urothelial carcinoma (BLCA, 7.11%), CHOL (11.11%), SARC (8.3%), and liver hepatocellular carcinoma (LIHC, 8.17%). We did not find high-level METTL3 amplifications above 5% in any TCGA tumor type (Fig. 1B, Supplementary Table S3).

For deep deletion of METTL genes in individual TCGA tumor types, only *METTL24* showed deep deletion rates above 5% in diffuse large B-cell lymphoma (DLBC, 10.41%), prostate cancer (PRAD, 8.38%), and uveal melanoma (UVM, 6.25%) (Fig. 1C, Supplementary Table S4). For somatic mutations, only *METTL4* exhibited somatic mutations in more than 5% of DLBC samples (Fig. 1D, Supplementary Table S5). Taken together, several METTL genes, including *METTL1*, *METTL2A*, *METTL4*, *EEF1AKNMT*, and *METTL24*, had relatively higher frequencies of genetic amplification, deletion, or mutation in various tumor types, notably LUAD, BRCA, and GBM.

### Analysis of METTL mRNA and protein expression in tumor and normal tissues

**Figure 2:**
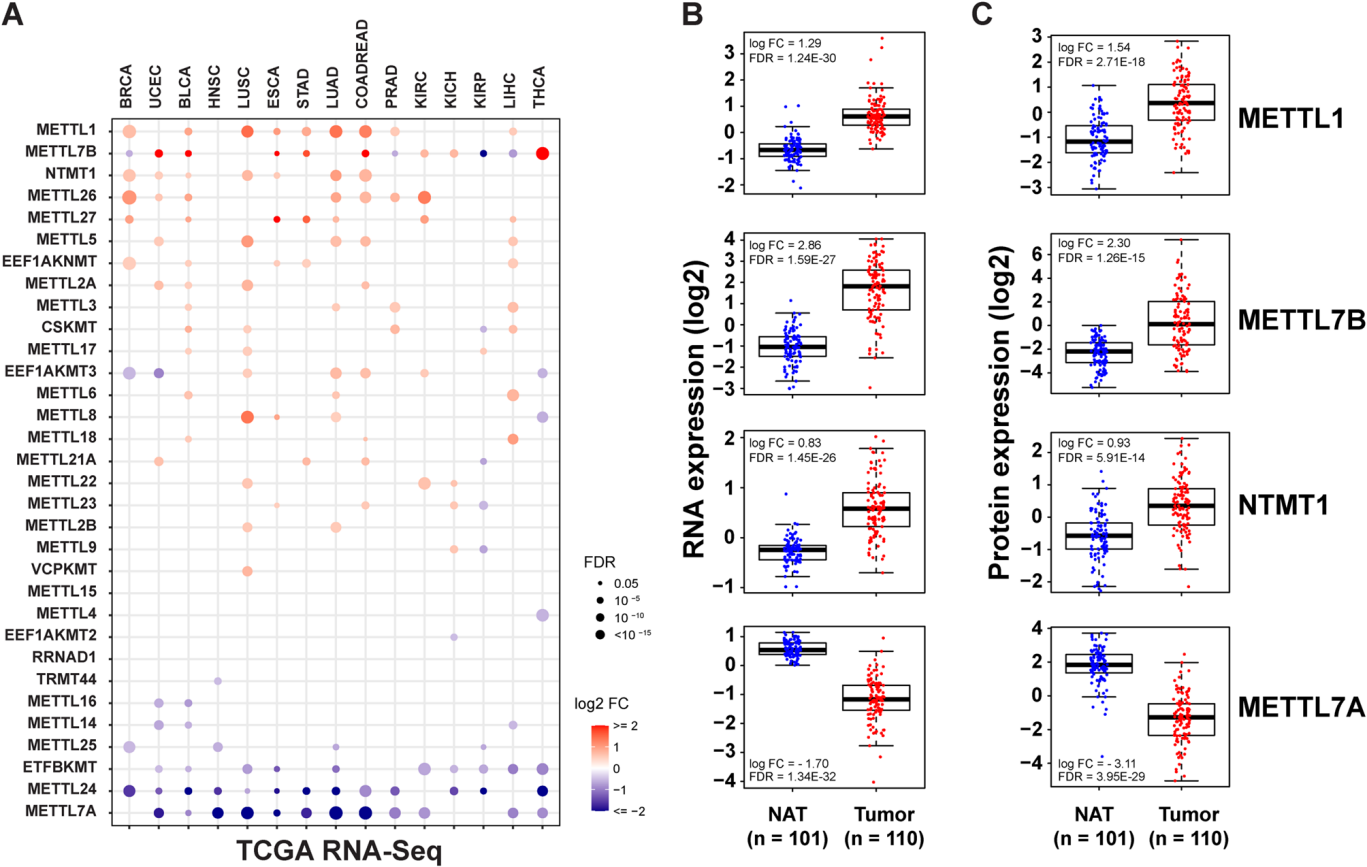
METTL mRNA and protein expression in TCGA and CPTAC samples. (**A**) Expression difference (log2 FC) for METTLs between TCGA tumor and normal samples. Only cancer types (15 TCGA tumor types) with more than 10 paired tumor-normal samples are shown. The color gradient from purple to red represents the degree of fold-change between tumor and normal. The dot size indicates the significance. The dot was filtered by fold change (log FC >|0.5|) and significance (FDR < 0.05). Boxplots showing (**B**) mRNA and (**C**) protein difference in four METTLs (METTL1, METTL7A, METTL7B, and NTMT1) in CPTAC–LUAD tumor and NAT samples. Blue: normal adjacent tissues (NAT); red: LUAD tumor samples. Sample numbers are indicated.

Comparing differentially expressed genes between tumor and paired-normal samples is critical for understanding the functional roles of METTL genes and guiding therapeutic discovery. Thus, we performed an integrative analysis of METTL mRNA and protein expression across tumor and normal samples in available TCGA and CPTAC datasets^13,15^. First, we determined changes in mRNA expression for 32 METTL genes in tumors relative to normal tissues for 15 TCGA tumor types with at least 10 normal samples available (Supplementary Table S2). We excluded *METTL11B* and *METTL21C* from these analyses due to their low mRNA expression level [RSEM (RNA-Seq by Expectation Maximization) < 1] in most TCGA samples; both genes are lineage-specific, expressing only in cardiomyocytes (*METTL11B*) or epididymis and skeletal muscle (*METTL21C*) based on the Human Protein Atlas database^16^. Out of the 32 METTLs, 10 showed significant [log2 fold change (FC) >|1|, false discovery rate (FDR) < 0.05] mRNA level changes in at least one tumor type (Fig. 2A, Supplementary Table S6, S7). Notably, three METTLs (*METTL1*, *METTL7B*, *METTL27*) were over-expressed in at least three of 15 TCGA tumor types compared to normal samples (Fig. 2A, Supplementary Tables S6, S7). For example, we found that *METTL1* was significantly overexpressed in LUAD, lung squamous cell carcinoma (LUSC), esophageal carcinoma (ESCA), and colorectal adenocarcinoma (COADREAD) samples compared to normal samples. In contrast, three METTLs (*METTL7A*, *METTL24*, *ETFBKMT*) were under-expressed (log2 FC <  − 1, FDR < 0.05) in at least three of 15 TCGA tumor types compared to normal samples (Fig. 2A, Supplementary Tables S6, S7). Notably, *METTL24*, the gene with the most substantial genetic deletions in TCGA tumor samples, showed significant mRNA downregulation in 11 TCGA tumor types, including BRCA and COADREAD (Fig. 2A, Supplementary Tables S6, S7).

Next, we compared METTL protein levels in six tumor types [LUAD, BRCA, OV, UCEC, CORD, ccRCC (clear cell renal cell carcinoma)] versus normal adjacent tissue (NAT) samples that were available as part of the CPTAC project in December 2020^15^. First, using the available RNA-seq data from more than 100 tumor-normal paired CPTAC–LUAD samples, we confirmed our findings in the TCGA–LUAD cohort^17^. We found that *METTL1*, *METTL7B*, and *NTMT1* were overexpressed, while *METTL7A* and *METTL24* were under-expressed in the CPTAC–LUAD tumors compared to NATs (Fig. 2B, Supplementary Fig. S2A). *METTL1* was upregulated in LUAD tumors compared to NATs with a log2 FC of 1.29 and FDR < 0.001 (Fig. 2B).

Next, we analyzed and compared METTL protein abundance in CPTAC proteomic data^15,17,18^. Approximately 5000–10,000 proteins were relatively quantified in multiple CPTAC tumor types^15,17,18^. Among 34 METTL proteins, 22 were identified and quantified in at least one of six CPTAC tumor types. We again found that a small number of METTLs, including METTL1 and METTL2A, were significantly elevated, while METTL7A was significantly decreased at the protein level in tumor tissue compared to NATs. (Fig. 2C, Supplementary Fig. S2B, Table S8).

Next, we analyzed the correlation between METTL DNA copy number, mRNA, and protein levels in CPTAC–LUAD tumor samples. We found that several METTLs, including METTL1, had a significantly positive correlation between DNA copy number, mRNA, and protein levels (Spearman *rho* > 0.5, FDR < 0.001, Supplementary Fig. S3, Tables S9, S10), suggesting that increasing DNA copy number contributed to increased METTL1 mRNA and protein levels in a subset of cancers.

### Identification of clinically relevant METTLs in human cancer

**Figure 3:**
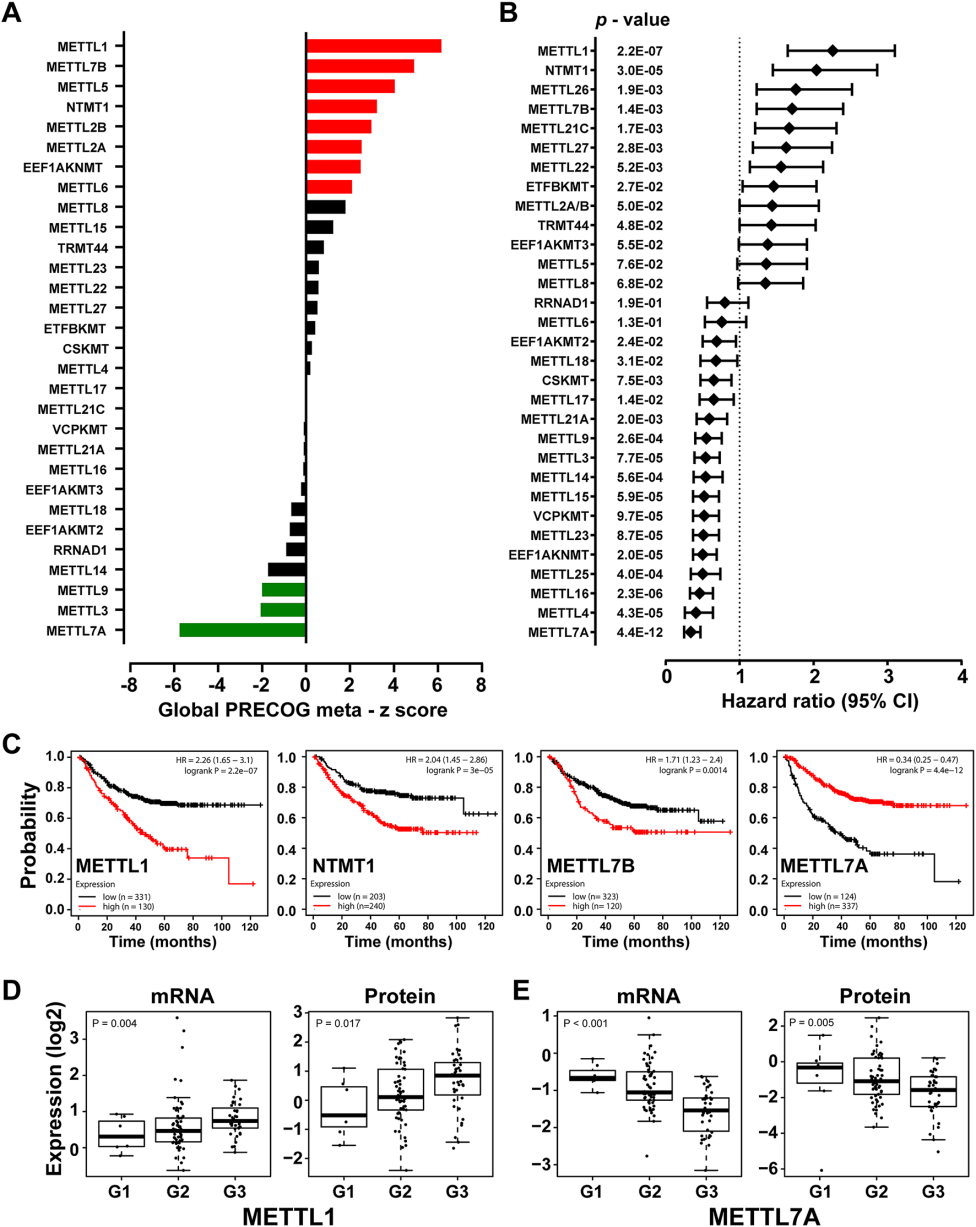
Prognostic roles of METTL family members in human cancer. (**A**) PRECOG meta z-scores for 30 METTL proteins in multiple cancer types. PRECOG z-score is a measurement of statistical significance with |1.96| equivalence to FDR < 0.05. Statistically significant positive z-score and adverse prognostic association (red). Statistically significant negative Z-score and favorable prognostic association (green). (**B**) Forest plot showing Univariate Cox regression analysis of 31 METTLs’ mRNA expression associated with LUAD patients’ progression-free survival: *HR* hazard ratio, *CI* confidence interval. (**C**) Kaplan–Meier progression-free survival curves for four METTLs’ (METTL1, NTMT1, METTL7B, and METTL7A) mRNA expression in LUAD patients. Boxplots showing mRNA and protein expression levels of (**D**) METTL1 and (**E**) METTL7A in three grades of CPTAC–LUAD samples. G1: Grade I, G2: Grade II and G3: Grade III.

Next, we investigated whether expression levels of METTLs were associated with patient survival in cancer. We first performed a meta-analysis of expression signatures from about 18,000 human tumors with survival outcomes using PRECOG (Prediction of Clinical Outcomes from Genomic Profiles)^19^. PRECOG z-scores, a measurement of statistical significance with |1.96| equivalent to FDR < 0.05, were obtained across different cancer types from the PRECOG website. This z-score encodes directionality of the association; a positive z-score indicates an adverse prognostic association, whereas a negative z-score indicates a favorable association. Overall, survival data for 30 of 34 METTL genes were found in the PRECOG datasets. Of the 30 METTLs, expressions of eight (*METTL1*, *METTL7B*, *METTL5*, *NTMT1*, *METTL2A*, *METTL2B*, *EEF1AKNMT*, *METTL6*) were significantly (FDR < 0.05) associated with unfavorable overall survival across cancers (Fig. 3A, Supplementary Table S11). Markedly, *METTL1* was ranked as the top METTL gene with a global meta z-score of 6.16 and notable individual tumor scores in neuroblastoma (9.45), BRCA (3.78), BCLC (2.79), and LUAD (1.69) (Fig. 3A, Supplementary Table S11). A subset of METTLs, such as *METTL1*, *METTL7B*, and *NTMT1*, which had higher frequency of gene alterations/overexpression in various cancers and/or were associated with poor disease prognosis, might function as cancer-promoters. In contrast, high expression of three METTLs (*METTL7A*, *METTL3*, and *METTL9*) was significantly (global meta-Z <  − 1.96) associated with favorable overall survival across cancers (Fig. 3A, Supplementary Table S11). Among METTLs, *METTL7A* had the most favorable overall survival global meta-Z score (− 5.75). The tumor type with the most favorable *METTL7A* z-score was LUAD (z-score =  − 4.86) (Fig. 3A, Supplementary Table S11).

Next, we focused on LUAD and analyzed whether expression of METTLs was associated with cancer progression and survival. LUAD was chosen due to its significant impact on global cancer-related mortality as well as several METTLs being genetically altered and/or upregulated in LUAD (Figs. 1, 2)^20^. For these analyses, we selected a LUAD cohort (461 LUAD samples with Affymetrix microarray data) with progression-free survival data in Kaplan–Meier Plotter ^21^. We found that high expression of *METTL1*, *NTMT1*, *METTL26*, and *METTL7B* was significantly associated with poor disease prognosis, while high *METTL7A* expression was associated with favorable progression in the LUAD cohort (Fig. 3B,C, Supplementary Fig. S4). We also analyzed METTL mRNA and protein expression across tumor grades in the CPTAC–LUAD cohort. Differences in mRNA and protein expression levels in METTL1 and METTL7A were observed according to LUAD tumor grade. METTL1 was highly expressed, while METTL7A was under-expressed in poorly differentiated, high-grade LUAD patients (Fig. 3D,E). In summary, transcriptomic and proteomic profiles of METTLs across a broad range of cancer types and their associations with clinical outcomes indicated that a subset of METTLs, such as METTL1, METTL7B, and NTMT1, might act as oncogenes, while METTL7A acts as a tumor suppressor.

### Proteogenomic landscape and functional dependency of METTLs in a larger cohort of cancer cell lines

Cancer cell lines are important model systems to study normal and aberrant cellular processes as well as biological functions of novel therapeutic targets^22–24^. First, we queried DNA copy number, mutations, and mRNA expression in more than 1000 CCLE (Cancer Cell Line Encyclopedia) lines^23^. We found that, similar to the TCGA Pan-Cancer cohort, at least ten METTLs (e.g. *METTL1*, *METTL2A*, *METTL2B*, *EEF1AKNMT*) showed high-level amplifications in more than 2% of CCLE lines (Supplementary Table S12). Nineteen METTLs exhibited deep deletion in more than 2% of CCLE lines, notably *METTL16* (11.85%) and *METTL24* (7.37%). Additionally, eight METTLs showed somatic mutations in more than 2% of CCLE lines, with *METTL16* in 3.31% and *TRMT44* in 3.12% of the samples (Supplementary Table S12). No METTL1 mutations were found in 1,570 CCLE lines^23^. Additionally, we performed qRT-PCR assays, and revealed that *METTL1* was highly expressed in several cancer lines, such as the breast cancer line MCF7 and the lung cancer line A549 (Supplementary Fig. S5).

**Figure 4:**
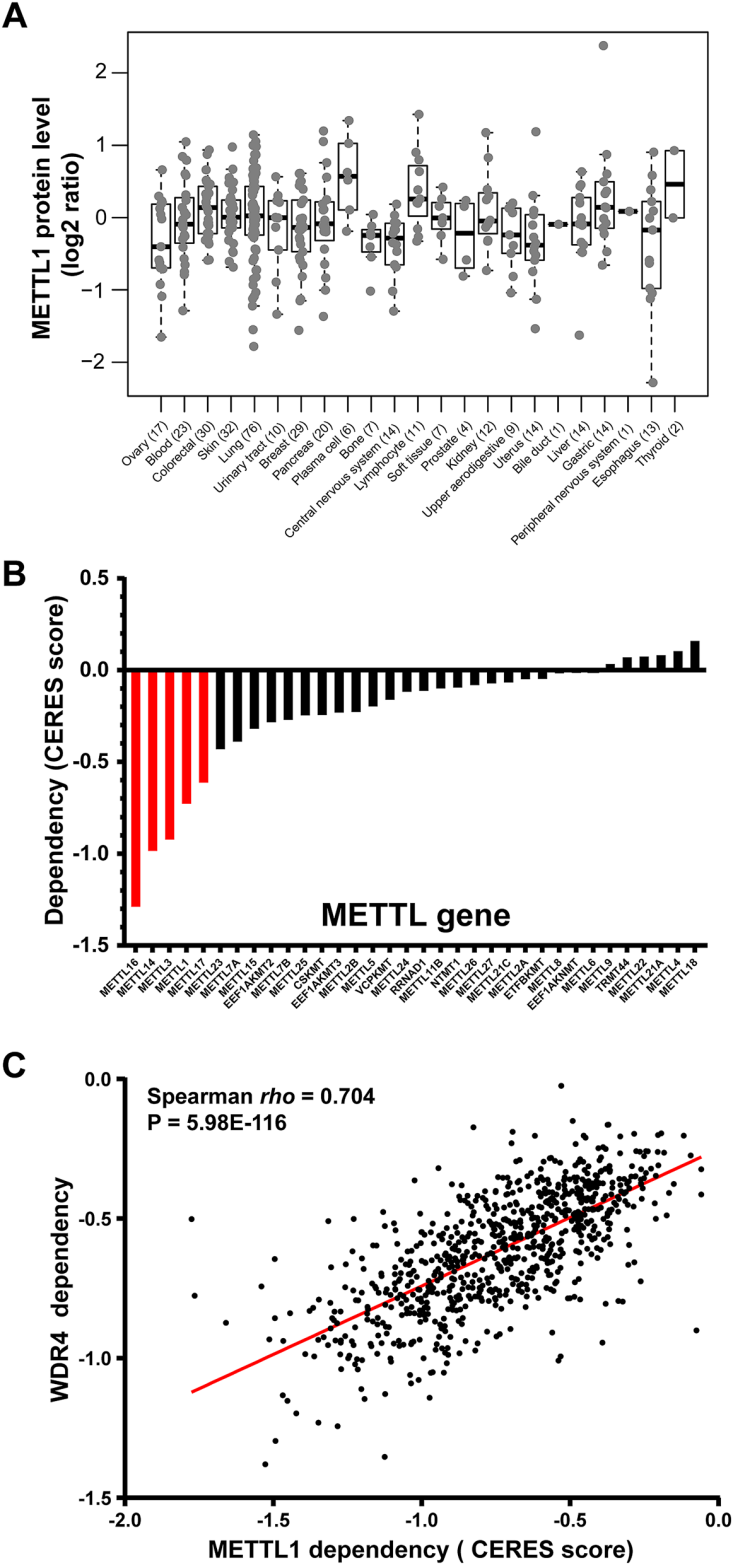
Proteogenomic landscape and functional dependency of METTLs in cancer cell lines. (**A**) Relative protein abundance of METTL1 in more than 300 CCLE lines across 22 lineages. (**B**) Bar graph showing mean of each METTL dependency score in genome-scale loss-of-function screens of 808 tumor lines. (**C**) Distribution of METTL1 dependency CERES score and its correlation with its cofactor WDR4 in 789 CCLE lines.

Recently, quantitative proteomics of 375 CCLE lines were profiled by mass spectrometry^24^. Analysis of proteomic profiling revealed that 15 METTL proteins were quantified in more than 50% of 375 tumor lines^24^. Figure 4A and Supplementary Fig. S6 show the relative protein abundance of METTL1, METTL7B, and NTMT1 in more than 300 CCLE lines across 22 lineages. In 11 CCLE lines, including two lung cancer lines NCIH1975 and CORL23, METTL1 had normalized log2 ratio greater than 1.0 (Fig. 4A)^24^.

The genome-wide loss-of-function screens of cancer cell lines with a CRISPR-Cas9 approach facilitates the interrogation of gene function^22^. To investigate the functional roles of METTLs, we evaluated their genetic vulnerabilities using data from the Cancer Dependency Map Project (DepMap)^22^. Average gene essentiality scores (CRISPR-Cas9 gene knockout scores [CERES]) that reflect gene dependence were calculated in 808 CCLE cell lines (20Q4 data) and the genes below a score of − 0.6 were retained^25^. We found that five METTLs, including *METTL1, METTL3, METTL14, METTL16*, and *METTL17*, showed lower than average CERES scores (< − 0.6) (Fig. 4B,C). Focusing specifically on the lung lineage, we found that 13 of 82 NSCLC (Non-small-cell lung carcinoma) lines exhibited an METTL1 CERES score of less than − 1.0^22^. In summary, loss-of-function screens of cancer cell lines support the biological importance of several METTLs, notably *METTL1, METTL3, METTL14, METTL16* and *METTL17*, in promoting cancer cell growth and survival.

### Functional annotation and pathway analysis of METTL1-associated proteins

**Figure 5:**
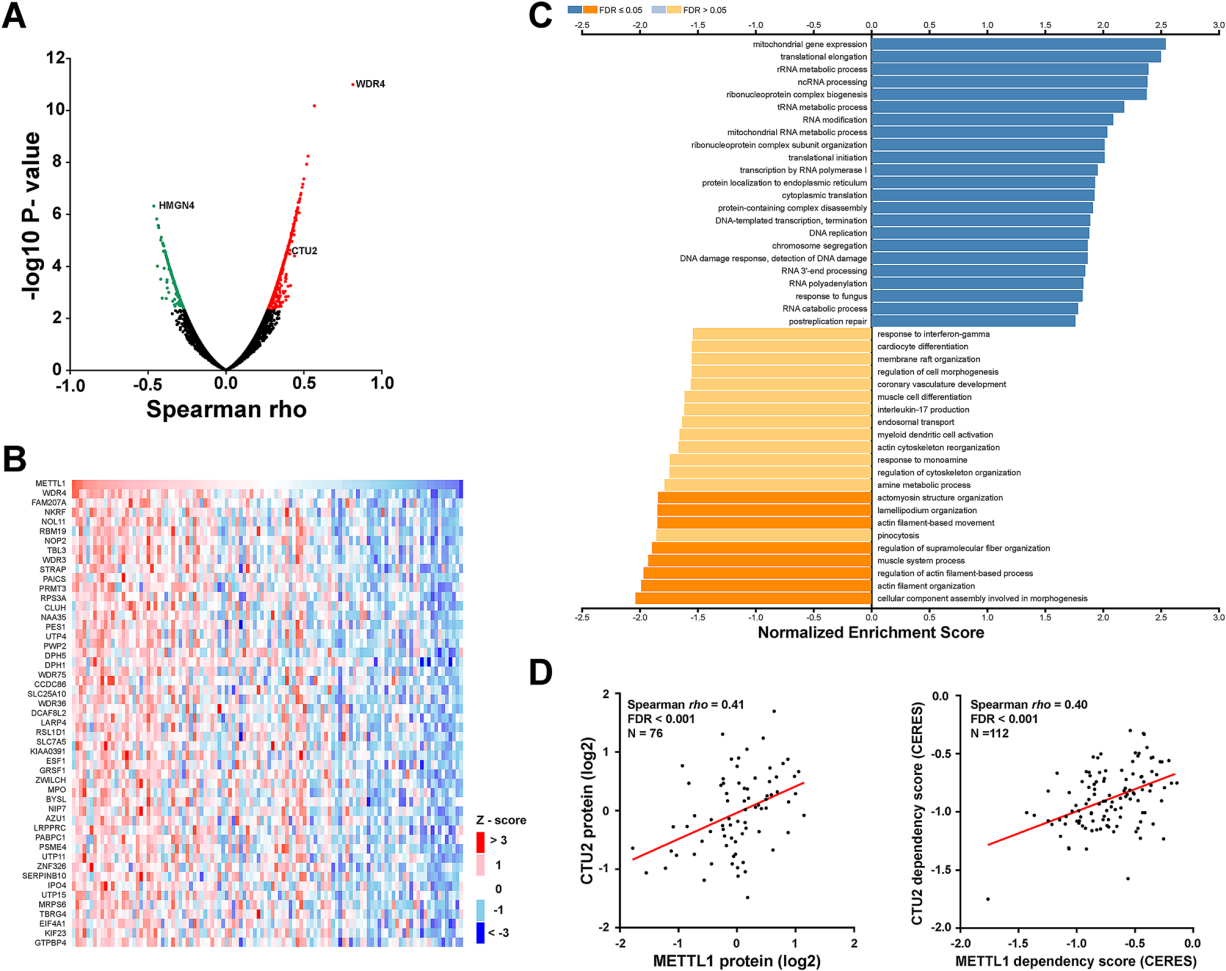
Functional interconnections and biological pathways of METTL1 in lung cancer. (**A**) Volcano plot showing abundance of protein having statistically significant positive (red dots) and negative (green dots) correlations with METTL1 protein abundance in CPTAC–LUAD cohort (Spearman correlation, FDR < 0.05). (**B**) Heatmap showing top proteins positively correlated with METTL1 protein in LUAD. (**C**) Gene Ontology of METTL1 correlated proteins in LUAD. Normalized enrichment scores were calculated with Gene Set Enrichment Analysis (GSEA) tool. (**D**) Correlations between the protein expression as well as cancer cell dependency scores of METTL1 and CTU2 in CCLE lung cancer lines.

Recent deep proteomic profiling of CCLE lines revealed that the primary variation in protein expression for most cell lines is organized by coordinated expression of protein complexes and cellular pathways^24^. METTL1 is an RNA methyltransferase that targets tRNA, mRNA, and miRNA. We hypothesized that METTL1 modulates many aspects of RNA metabolism, influences protein synthesis rate, and has numerous functional effects on cellular pathways and cancer progression. Thus, we chose METTL1 as a candidate for further investigation of its expression correlation network and pathways in human cancer. Three proteomics datasets, CPTAC–LUAD, CPTAC–BRCA, and CCLE, were selected because they quantified more than 10,000 proteins each^26^. The function module of LinkedOmics was applied to analyze proteomic data from 110 LUAD and 122 BRCA samples in the CPTAC^27^. As shown in the volcano plot (Fig. 5A), 726 proteins (red dots) showed significantly positive correlations with METTL1, whereas 286 proteins (green dots) showed significantly negative correlations (Spearman correlation, FDR < 0.05) in CPTAC–LUAD. As expected, the METTL1 essential cofactor WDR4 was the top protein showing a statistically significant positive correlation with METTL1 in the CPTAC–LUAD cohort (Spearman *rho* 0.81, FDR 1.00E−07) (Supplementary Fig. S7). The top 50 genes are shown in the heatmap (Fig. 5B). To further analyze the biological functions of METTL1–correlated proteins in LUAD, we applied the Gene Set Enrichment Analysis (GSEA) in the LinkInterpreter module of LinkedOmics to calculate the normalized enrichment scores (NES) of Gene Ontology (GO) biological process with FDR ranking and 1,000 simulations. As shown in Fig. 5C, METTL1–correlated proteins are enriched in various RNA modification and metabolic processes, translational regulation, and transcription.

To identify common proteins functionally associated with METTL1 in various tumors, we performed a similar analysis of proteomics datasets of CPTAC–BRCA and CCLE. Next, we merged the three datasets together into a common dataset. Using a cutoff value of FDR < 0.05, a total of 187 proteins showed significant correlation with METTL1 protein expression in three cohorts. Among them, 115 demonstrated consistently positive correlation, while only 11 demonstrated consistently negative correlation with METTL1 across all 3 datasets. When a more stringent cutoff value (FDR < 0.01) was used, 40 proteins were retained. Of those, 35 were consistent across all three datasets, 34 being positively correlated with METTL1. These positively correlated proteins include METTL1–essential cofactor WDR4 and other RNA metabolism regulators (XPOT, NSUN2, TRMT6) that may function coordinately with METTL1 (Supplementary Table S13). We also revealed that all five members of the condensin I complex (NCAPD2, NCAPG, NCAPH, SMC2, SMC4), which are involved in chromosomal segregation during mitosis and meiosis, were included as part of this protein cohort (Supplementary Table S13). We hypothesize that some of these proteins may be METTL1 direct downstream targets. Only one protein, HMGN4 (high mobility group nucleosomal binding domain 4), demonstrated a significantly and consistently negative correlation with METTL1 protein levels in all three cohorts. Notably, CRISPR–Cas9 knockout screening of HMGN4 in CCLE lines yielded a positive score, suggesting tumor suppressing functions of HMGN4 (Data not shown).

Recent studies demonstrate that integrating CRISPR–Cas9 screens of diverse cancer cell lines can generate a map of genetic interactions and identify network modules with similar functional characteristics^28^. Accordingly, we also computed the correlation between CERES score of METTL1 and other genes in CRISPR/Cas9 screens of 808 tumor lines. The top three genes positively correlated with METTL1 in CRISPR/Cas9 CCLE screens were WDR4, ADAT2 (adenosine deaminase tRNA specific 2), and TRMT61A (tRNA methyltransferase 61A) (Fig. 4C). In addition, two of the 187 proteins significantly correlated with METTL1 protein expression, CTU2 (cytosolic thiouridylase subunit 2) and XPOT (exportin for tRNA), also exhibited significant positive correlation with the METTL1 CRISPR/Cas9 CERES score in CCLE lung cancer cell lines (Fig. 5D). It is worthwhile to determine crosstalk and functional roles between METTL1, CTU2, and XPOT in human cancer, notably lung cancer.

### Structural analysis and modelling of METTL1–WDR4 complex

Structural studies of methyltransferase ligand/protein complexes provide insight into the catalytic mechanism and inform the discovery of potential inhibitor for therapeutic applications. A high-resolution crystal structure (3D structure) of the METTL1 enzymatic domain (37−265aa) was solved in complex with SAM, which is publicly available in RCSB Protein Data Bank (PDB: 3CKK, Resolution: 1.55 Å). Accordingly, we first examined the druggability of the SAM binding site of METTL1 (PDB: 3CKK) with the DoGSiteScorer tool^29^. With values between zero and one (higher score indicates higher druggability), the SAM pocket has the relatively high drug score (0.79) (Supplementary Fig. S8). METTL1 is conserved from yeast to mammals; primary amino acid sequence of METTL1’s enzymatic domain (77–254 aa) was found to be 64.04% identical to yeast Trm8 (77–281 aa) (Supplementary Fig. S9). Additionally, the structure of Trm8−Trm82 heterodimer has been solved (PDB: 2VDU; Resolution: 2.4 Å) and the RNA binding model of Trm8–Trm82 was generated based on a small-angle X-ray scattering approach^30^.

**Figure 6:**
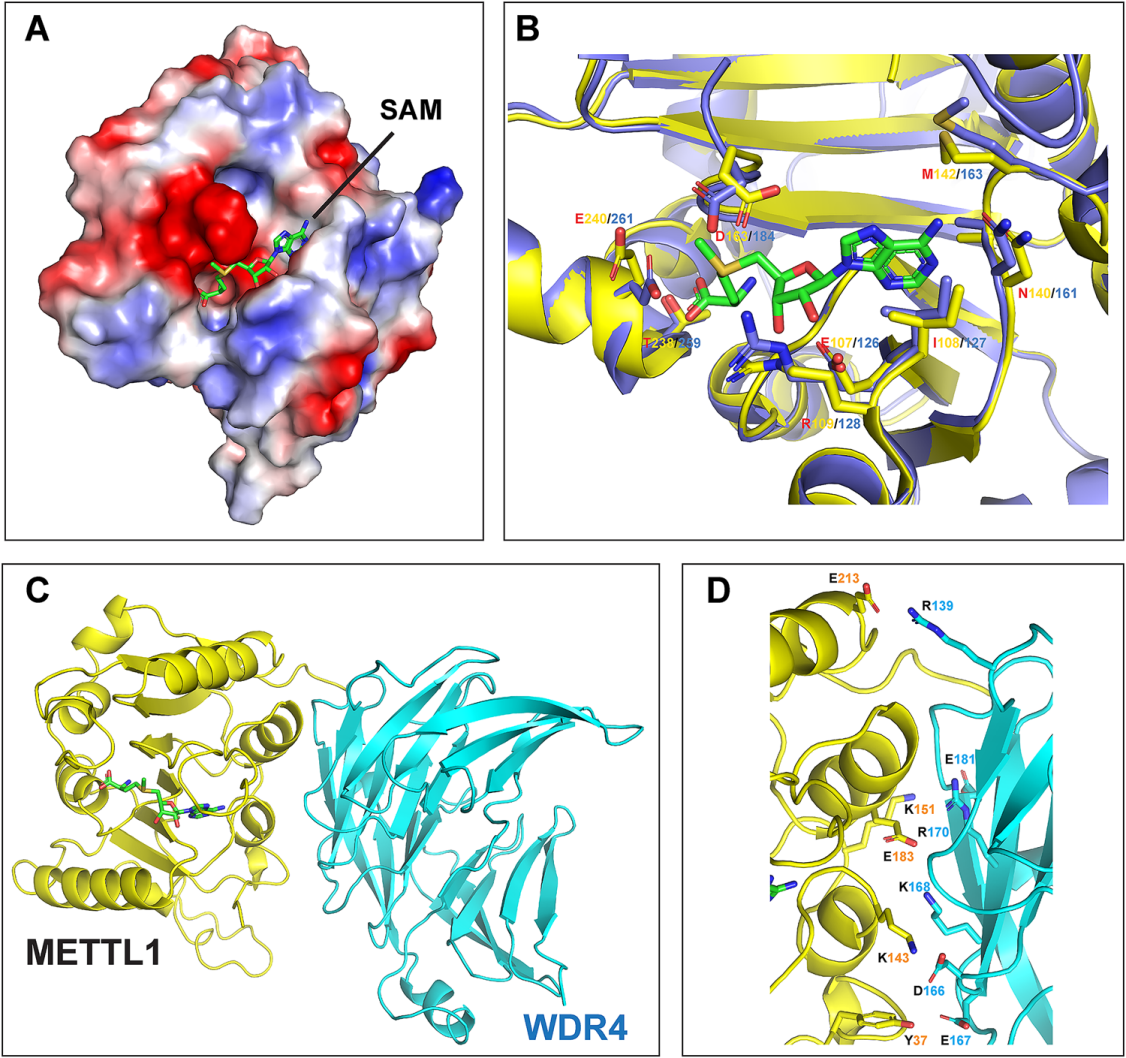
Modelling structure of METTL1–WDR4. (**A**) Surface representation of the SAM binding pocket in METTL1 with color according to the electrostatic potential. The surface electrostatic potential diagram (± 5 kT/e) in METTL1 (PDB: 3CKK) was generated by PyMol; the blue areas represent positively charged areas, while the red areas represent negatively charged areas. (**B**) Superposition of the key residues of SAM binding pockets of human METTL1 (PDB: 3CKK) and Yeast Trm8 (PDB: 2VDU). (**C**) Graphic representation of the METTL1–WDR4 heterodimeric complex model. (**D**) Close-up view of the interaction between METTL1 and WDR4 with the key residues labeled.

Next, we analyzed the overall structure and key functional residues of human METTL1 in detail. The METTL protein has an expected Rossmann fold built around a β sheet containing seven strands in the order β3β2β1β4β5β7β6. The overall conformation of the SAM binding pocket of METTL1 is similar to that of yeast Trm8 as most of the residues constituting the binding pocket are well conserved (Fig. 6A,B, Supplementary Fig. S9). The consensus GxGxG motif (G84, G86, and G88 in METTL1) lies at the bottom of the pocket. D163 and E240 help position the methyl group of SAM to the m^7^G binding pocket that is adjacent to the SAM binding site (Fig. 6B). When compared to Trm8 3D structure, the surface groove that connects the m^7^G binding pocket and SAM binding pocket adopts a more open shape in METTL1 structure compared with that of Trm8 due to different side chain conformations of R109, D163, and E240. E109 and G86 form hydrogen bonds with the ribose of SAM. The methionine moiety is stabilized through multiple interactions, including hydrogen bonds with E107, I108, N140, A141, and M142, hydrophobic interaction with I108, and salt bridge with E107. In summary, METTL1 has the class I Rossmann fold as well as highly conserved catalytic residues, and SAM-binding pocket.

WDR4 is the essential co-factor of METTL1; inhibitors targeting METTL1–WDR4 interaction are also promising. Based on the Trm8–Trm82 complex structure, we generated the 3D model of METTL1–WDR4 complex using homology modeling in SWISS-MODEL servers, and molecular simulations with Rosetta^31–36^. Figure 6C illustrates the overall structure of the METTL1–WDR4 complex. Based on this model, we found that WDR4 interacts using a core of three residues (D166, E167, and K168) which form hydrogen bonds with K143 and Y37 of METTL1 (Fig. 6D). Additionally, R139 and E181 of WDR4 form hydrogen bonds with E213 and K151 of METTL1, respectively (Fig. 6D). Furthermore, K168 and R170 of WDR4 form salt bridges with E183 of METTL1 (Fig. 6D). The interaction between WDR4–R170 and METTL1–E183 is speculated to be critical for the activation of METTL1. Studies show that primordial dwarfism patients with the WDR4–R170L missense mutation have defects in m^7^G levels in tRNA^37^. Even though further research is needed to deeply understand the structure and biochemical properties of the METTL1–WDR4 complex, our primary structure analysis provides a clue of key residues that are likely critical for METTL1 methyltransferase function.

## Discussion

In this study, we performed integrated genomic, transcriptomic, proteomic, and clinicopathological analyses of 34 METTLs in a large cohort of primary tumors and cell lines. We identified a subset of METTL genes, notably METTL1, METTL2A, METTL2B, METTL7B, NTMT1, and METTL26, with high frequencies of genomic amplification and/or up-regulation, while METTL7A and METTL24 were under-expressed, at both mRNA and/or protein levels in a spectrum of human cancers. We revealed that expression of a subset of METTLs, particularly METTL1, was associated with high-grade tumors and poor disease prognosis, particularly in LUAD. Loss-of-function analysis in large cohorts of tumor cell lines indicated the biological importance of METTL1, an m^7^G methyltransferase, in cancer cell growth and survival. Furthermore, functional annotation and pathway analysis of METTL1-associated proteins revealed that, in addition to METTL1 cofactor WDR4, two tRNA regulators, CTU2 and XPOT, may be functionally interconnected with METTL1 in human cancer. Finally, using molecular modeling and simulation approaches, we generated a 3D model of METTL1–WDR4 that might aid in understanding the key functional residues and the development of small inhibitors.

Even though all 34 METTL proteins contain a conserved methyltransferase-like domain, they have different subcellular localizations. Based on COMPARTMENTS (subcellular localization database), several METTLs (e.g. METTL3, METTL5, METTL14, and METTL16) are primarily nuclear; METTL1, EEF1AKMT2, and NTMT1 localize to the nucleus and cytosol; METTL4, METTL15, and METTL17 localize to the nucleus and mitochondrion; ETFBKMT (METTL20) is primarily localized to the mitochondrion; and METTL7A is primarily localized to the endoplasmic reticulum^38^. It is plausible that METTL proteins perform various biological functions in multiple compartments of the cell. For example, METTL3 and its cofactor METTL14, two well-studied METTLs, catalyze m^6^A methylation of mRNA or non-coding RNA in mammals^39^. Many recent studies revealed METTL3’s involvement in pathways affecting cell proliferation, cell death, invasion, and metastasis in cancer^39^. Nevertheless, using the TCGA and CPTAC tumor dataset, we did not observe substantial genetic alterations or upregulations of METTL3 and METTL14 in cancer. However, the loss-of-function analysis in larger cohort of tumor cell lines indicated the biological importance of METTL3 and METTL14 in cancer cell growth and survival (Fig. 4B).

In this study, we found that several METTLs, such as METTL2A, METTL2B, and METTL26, which have not been previously investigated systematically, are upregulated at mRNA and protein levels in a subset of human cancers compared to normal tissue. METTL2A and METTL2B form a complex that is involved in m^3^C methylation at position 32 of the anticodon loop in certain tRNAs^40^. A recent study revealed that human METTL2A and METTL2B form a complex with the DALRD3 (DALR anticodon binding domain containing 3) protein to recognize particular arginine tRNAs^41^. To date, METTL26 has been sparsely studied; its substrate remains unknown and current reports of METTL26 only discuss it in terms of exon skip alternative splicing in cancer^42–45^. Our pathway analysis of METTL26-associated proteins in CPTAC and CCLE datasets indicated that METTL26 might be involved in the vacuolar transport in human cancer (Data not shown). Additionally, our study revealed that NTMT1, which methylates the α-N-terminal amines of proteins [e.g., RCC1 (regulator of chromosome condensation 1), CENPA (centromere protein A), and DDB2 (damage specific DNA binding protein 2)], likely possesses cancer-promoting roles. Interestingly, inhibitors targeting NTMT1 have been reported and biochemically characterized^46,47^. It would be interesting to test cellular impacts of these NTMT1 inhibitors on cancer cells in the future.

One interesting finding of our current study is the potentially opposing roles of two close homologues, METTL7A and METTL7B, in cancer aggressiveness and progression. METTL7A is under-expressed, while METTL7B is over-expressed in TCGA and CPTAC tumors compared to normal tissue (Fig. 2). METTL7A is reported as an integral membrane protein anchored into the endoplasmic reticulum membrane and has roles in lipid droplet formation^48,49^. In vitro and in vivo functional studies employing overexpression and knockdown cell models reveal METTL7A as a novel tumor suppressor in liver cancer^50^. On the other hand, multiple studies indicated that METTL7B functions as an oncogene. In breast cancer, siRNA silencing of METTL7B dramatically inhibits cancer invasion^51^. In lung cancer, overexpression of METTL7B significantly influenced tumor growth in vivo and in vitro^52^. A recent study claims that METTL7B is an alkyl thiol methyltransferase that methylates hydrogen sulfide residues and has potential to alter the redox state and growth cycle of cells^53^.

Our studies, together with others, strongly support the oncogenic roles of METTL1 in various cancers^4,5,54–56^. Tian et al*.* reported that METTL1 is upregulated in liver cancer and exhibits oncogenic activities via the PTEN/AKT signaling pathway^5^. Liu et al. reported that combined knockdown of METTL1 and NSUN2 increases HeLa cell sensitivity to 5-fluorouracil via tRNA destabilization^4^. Furthermore, in this study, we also revealed that two tRNA regulators, CTU2 and XPOT, may be functionally interconnected with METTL1 in cancer. CTU2 forms a complex with CTU1, which plays a role in thiolation of uridine residues present at the wobble position in a subset of tRNAs, resulting in enhanced codon reading accuracy^57^. Recently, Rapino et al*.* reported that CTU1/CTU2 are key players in protein synthesis rewiring that is induced by the transformation driven by the BRAF oncogene mutation and by resistance to targeted therapy in melanoma^58^. XPOT, a member of RAN-GTPase exportin family, which mediates export of tRNA from the nucleus to the cytoplasm, promotes tumor proliferation and invasion in liver cancer^59^. Thus, METTL1 likely forms a functional network and regulates various RNA methylation events and pathways, promoting cancer progression.

Human methyltransferases, such EZH2, PRMT5, and DOT1L, are being actively pursued as drug targets for various cancers. Furthermore, inhibitors targeting METTL family members, such as METTL3 and NTMT1, were also identified and characterized recently^46,60^. However, no METTL1 inhibitors have been reported to date. In current study, we analyzed the druggability of METTL1 SAM-binding pocket and revealed key functional residues of METTL catalytic mode. More importantly, analysis of the METTL1–WDR heterodimeric complex identified key residues (WDR4–R170 and METTL1–E183) that likely have critical roles for the methyltransferase function of METTL1. Recent studies have demonstrated that several RNA modification enzymes require partner proteins, e.g., METTL3–METTL14, METTL5–TRMT112 (tRNA methyltransferase activator subunit 11-2)^61^. Our current proteomic and loss-of-function analysis also indicated the essential roles of WDR4 in METTL1’s biological function in human cancer. Thus, inhibitors targeting METTL1–WDR4 interface, notably the interaction between WDR4–R170 and METTL1–E183, are worth pursuing.

In summary, we employed an integrated multi-omics approach to identify critical METTLs in human cancer; inhibitors targeting these METTLs have therapeutic potential in certain cancer types. However, it should be noted that future studies are needed to address the pathophysiological significance and molecular mechanisms of the identified METTLs, such as METTL1, NTMT1, and METTL26, in promoting cancer development and progression.

## Materials and methods

### Copy number and mutational analysis of METTL genes in TCGA tumors

Genetic alteration data from 10,967 tumor samples covering 32 tumor types in The Cancer Genome Atlas (TCGA) Pan-Cancer studies were obtained from the cBio Cancer Genomics Portal (http://www.cbioportal.org). In cBioPortal, the copy number for each METTL gene was generated by the GISTIC algorithm and categorized as copy number level per gene: − 2 is considered a possible homozygous deletion, − 1 is considered a heterozygous deletion, 0 is considered diploid, − 1 is considered a low-level gain, and 2 is considered a high-level amplification. DNA copy number and mutations in more than 1000 CCLE (Cancer Cell Line Encyclopedia) lines were also obtained from cBioPortal^14^. Heatmaps were generated using the Morpheus online software suite (https://software.broadinstitute.org/morpheus/).

### Analysis of METTL mRNA expression in TCGA and CPTAC tumor and normal samples

Normalized RNA-sequence data in 11,069 TCGA samples, including tumor and 737 normal samples, was downloaded from GDC portal (https://gdc.cancer.gov/about-data/publications/pancanatlas; File: EBPlusPlusAdjustPANCAN_IlluminaHiSeq_RNASeqV2.geneExp.tsv). Tumor types that contain at least 10 paired TCGA normal samples were selected to calculate the mRNA difference between tumor and normal samples. Normalized RNA-sequence data in CPTAC–LUAD dataset was downloaded from the original article^17^. The Wilcoxon Rank-Sum test, with FDR calculated via the Benjamini–Hochberg procedure was applied using the R software (https://www.R-project.org). Bubble, dot, and boxplots were generated with ggplot2 and reshape2 packages in R.

### Analysis of METTL protein expression in tumor and normal samples

Normalized proteomic data (log2 ratio) from six CPTAC datasets [LUAD, BRCA, OV, UCEC, COAD, ccRCC (clear cell renal cell carcinoma)] and their clinical data were downloaded from CPTAC Portal (https://cptac-data-portal.georgetown.edu), LinkedOmics (www.linkedomics.org), or original published papers^17,18,27,62–65^. The CPTAC project used a standard protocol to collect and analyze data from different tumor types^15^. Quantitative proteomics of 375 CCLE lines were downloaded from original published data and DepMap portal (https://depmap.org/portal)^24^. Statistical significance of the differences in protein expression levels for each METTL between tumor and NATs and between different tumor grades was determined using Wilcoxon Rank-Sum test and ANOVA. Spearman and Pearson correlation tests were used to correlate copy numbers, mRNA, and protein levels of each METTL from CPTAC–LUAD specimens. We used the ‘cor’ function in R for computation, specifying the appropriate statistical test (Spearman or Pearson).

### Survival analysis of METTL in cancer patients

To determine PRECOG z-scores for global and individual tumor overall survival in ~ 18,000 tumors, we searched for all genes in the METTL family by their official names in the PRECOG-meta-Z file; if those names were not present, alternative and previous symbols were also queried. Relationships between METTL mRNA expression and progression-free survival in LUAD were analyzed by dividing samples into high and low expression groups for each METTL based on “auto select best cutoff” in Kaplan–Meier Plotter (https://kmplot.com).

### Analysis of METTL1-associated proteins

The ‘cor’ function in R and the LinkedOmics function module were applied to analyze proteomic data from CCLE tumor lines, CPTAC–LUAD, and CPTAC–BRCA samples. The ‘cor’ function (Spearman and Pearson) in R was also used to compute the correlation between CERES score of METTL1 and other genes in CRISPR/Cas9 screens of 808 CCLE tumor lines. Gene Set Enrichment Analysis (GSEA) in the LinkInterpreter module of LinkedOmics was applied to calculate the normalized enrichment scores (NES) of Gene Ontology (GO).

### Structural analysis and modelling of METTL1–WDR4 complex

The primary amino acid sequences of human METTL1, WDR4, Yeast Trm8 and other related proteins were retrieved from the NCBI (National Center for Biotechnology Information) Database. The protein sequences were aligned using Clustal Omega (https://www.ebi.ac.uk/Tools/msa/clustalo/) and were presented with the ESPript 3.0 program (http://espript.ibcp.fr/ESPript/ESPript/). Crystal structures of human METTL1 and Yeast Trm8–Trm82 were obtained from RCSB Protein Data Bank and analyzed with PyMOL and Protein–Ligand Interaction Profiler programs^66,67^. To generate structural model of METTL1–WDR4 heterodimer, a 3D structure Trm8–Trm82 heterodimer was used as a template in the Swiss-Model homology modelling server^30,32^. Then the Rosetta program was used to simulate the 3CKK and SWISS-Model generated structures. Briefly, after orienting the two proteins in their expected positions based on Trm8–Trm82 heterodimer using PyMol, the structures were prepared with the Relax protocol of Rosetta^33,34^. This protocol alternated between sidechain packing and gradient-based minimization of torsional degrees of freedom. One cycle consisted of four rounds of the two optimizations, with each round increasing in repulsive contribution to total energy. Five cycles were performed before the most energetically favorable was selected as output. In addition, the protocol was instructed to constrain backbone heavy atom position based on starting structure. This entire procedure was done twice and the resulting model with the lesser total energy was used for the docking simulation. The docking was done in two stages^35,36^. First, the proteins were represented coarsely by replacing side chains with unified pseudo-atoms, or centroids. A 500-step Monte Carlo search with dynamically adjusting rotational and translational steps was performed with an acceptance rate of 25%. Second, the structure with the lowest energy underwent high-resolution refinement through 50 minimization steps. Each step perturbed the position of the proteins by a random Gaussian distribution of 0.1 Å and 3°, minimized the perturbed orientation’s energy, and optimized the side chains with rotamer trials, and the result was accepted or rejected based on the Metropolis criterion. Every eight steps, an additional side chain optimization and a Metropolis criteria check were done. Rotamer trials chose the single best rotamer at a random position in the context of the current state of the rest of the system, with the positions visited once each in random order. Each simulation started by perturbing the METTL1–SAM structure by a random Gaussian distribution of 3 Å and 8° before moving on to the coarse and fine stages. The simulation was repeated 100 times to produce 100 possible docked orientations. The orientation with the lowest energy was chosen as the structural model. Structure visualization and mapping of residues was performed using PyMOL^66,67^.

## Supplementary Information

Supplementary Information.

## Data Availability

All data generated or analyzed during this study are included in this published article (and its Supplementary Information files). All the original data used in this study are freely available on the websites or links provided in this article. The datasets and code used and/or analyzed during the current study are available from the corresponding authors on reasonable request.
