# Supplementary material for: Multi-omics integration of methyltransferase-like protein family reveals clinical outcomes and functional signatures in human cancer
Source: Sci Rep. 2021 Jul 20;11:14784. doi: 10.1038/s41598-021-94019-5 (PMC8292347; doi:10.1038/s41598-021-94019-5)
Supplement: Supplementary file 1 — Supplementary Information. [file 41598_2021_94019_MOESM1_ESM.pdf]

Supplementary Information

Multi-omics integration of methyltransferase-like protein family reveals clinical outcomes and functional signatures in human cancer

Ion John Campeanu[1, *], Yuanyuan Jiang[1, *], Lanxin Liu[1], Maksymilian Pilecki[1], Alvina Najor[1], Era Cobani[1], Morenci Manning[1], Xiaohong Mary Zhang[1, 2], and Zeng-Quan Yang[1, 2]

[1] Department of Oncology, Wayne State University School of Medicine, Detroit, MI, USA.
[2] Molecular Therapeutics Program, Barbara Ann Karmanos Cancer Institute, Detroit, MI, USA.

Corresponding author: Zeng-Quan Yang, Ph.D., Barbara Ann Karmanos Cancer Institute, 4100 John R Street, HWCRC 815, Detroit, MI 48201 Tel: (313)576-8339; Fax: (313)576-8029; E-mail: yangz@karmanos.org

Table S1. List of 34 human METTL genes

Gene Symbol	Full Name	Aliases	NCBI ID	Gene Location
METTL1	methyltransferase like 1	TRM8, TRMT8, C12orf1, YDL201w	4234	12q14.1
METTL2A	methyltransferase like 2A	FLJ12760, METTL2	339175	17q23.2
METTL2B	methyltransferase like 2B	METTL2, METL, FLJ11350, METTL2A, PSENIP1	55798	7q32.1
METTL3	methyltransferase like 3	Spo8, M6A, MT-A70, IME4, hMETTL3	56339	14q11.2
METTL4	methyltransferase like 4	FLJ23017, HsT661	64863	18p11.32
METTL5	methyltransferase like 5	HSPC133, MRT72	29081	2q31.1
METTL6	methyltransferase like 6	MGC24132	131965	3p25.1
METTL7A	methyltransferase like 7A	AAMB, DKFZP586A0522	25840	12q13.12
METTL7B	methyltransferase like 7B	MGC17301, ALDI	196410	12q13.2
METTL8	methyltransferase like 8	FLJ13984, TIP	79828	2q31.1
METTL9	methyltransferase like 9	DREV1, DREV, PAP1, CGI-81	51108	16p12.2
EEF1AKMT2	EEF1A lysine methyltransferase 2	METTL10, C10orf138, Efm4	399818	10q26.13
NTMT1	N-terminal Xaa-Pro-Lys N-methyltransferase 1	METTL11A, C9orf32, AD-003, HOMT1A, NRMT1, NRMT, NTM1A	28989	9q34.11
METTL11B	methyltransferase like 11B	C1orf184, HOMT1B, NTM1B	149281	1q24.2
CSKMT	citrate synthase lysine methyltransferase	METTL12, U99HG, CS-KMT	751071	11q12.3
EEF1AKNMT	eEF1A lysine and N-terminal methyltransferase	METTL13, KIAA0859, DFNM1, CGI-01, FEAT, DFNB26, DFNB26M, 5630401D24Rik	51603	1q24.3
METTL14	methyltransferase like 14	KIAA1627, hMETTL14	57721	4q26
METTL15	methyltransferase like 15	METT5D1, FLJ33979	196074	11p14.1
METTL16	methyltransferase like 16	METT10D, MGC3329	79066	17p13.3
METTL17	methyltransferase like 17	METT11D1, FLJ20859	64745	14q11.2
METTL18	methyltransferase like 18	C1orf156, MGC9084, AsTP2, HPM1	92342	1q24.2
TRMT44	tRNA methyltransferase 44 homolog	METTL19, C4orf23, FLJ35725, TRM44	152992	4p16.1
ETFBKMT	electron transfer flavoprotein subunit beta lysine methyltransferase	METTL20, C12orf72, DKFZp451L235, MGC50559, ETFB-KMT	254013	12p11.21
METTL21A	methyltransferase like 21A	FAM119A, LOC151194, HCA557b, HSPA-KMT	151194	2q33.3
EEF1AKMT3	EEF1A lysine methyltransferase 3	METTL21B, FAM119B, DKFZP586D0919	25895	12q14.1
METTL21C	methyltransferase like 21C	C13orf39, LOC196541	196541	13q33.1
VCPKMT	valosin containing protein lysine methyltransferase	METTL21D, C14orf138, VCP-KMT	79609	14q21.3
METTL22	methyltransferase like 22	C16orf68, FLJ12433, MGC2654	79091	16p13.2
METTL23	methyltransferase like 23	C17orf95, LOC124512, MRT44	124512	17q25.2
METTL24	methyltransferase like 24	C6orf186	728464	6q21
METTL25	methyltransferase like 25	C12orf26, FLJ22789	84190	12q21.31
RRNAD1	ribosomal RNA adenine dimethylase domain containing 1	METTL25B, C1orf66, CGI-41	51093	1q23.1
METTL26	methyltransferase like 26	C16orf13, MGC13114, JFP2	84326	16p13.3
METTL27	methyltransferase like 27	WBSCR27	155368	7q11.23

Table S2: TCGA Pan-Cancer tumor type and abbreviation

Tumor Type	Abbreviation	Total Tumor	Tumor Sequenced	Tumor CNA	Tumor RNA-Seq	Normal Tissue RNA-seq
**Breast invasive carcinoma**	BRCA	1084	1066	1070	1082	114
**Ovarian serous cystadenocarcinoma**	OV	585	523	572	300	
**Uterine Corpus Endometrial Carcinoma**	UCEC	529	517	523	527	34
**Uterine Carcinosarcoma**	UCS	57	57	56	57	
**Cervical squamous cell carcinoma and endocervical adenocarcinoma**	CESC	297	291	293	294	3
**Bladder Urothelial Carcinoma**	BLCA	411	410	408	407	19
**Head and Neck squamous cell carcinoma**	HNSC	523	515	517	515	44
**Lung squamous cell carcinoma**	LUSC	487	484	487	484	51
**Esophageal carcinoma**	ESCA	182	182	182	181	11
**Stomach adenocarcinoma**	STAD	440	436	438	412	35
**Colon & Rectum adenocarcinoma**	COAD/READ	594	534	592	592	51
**Pancreatic adenocarcinoma**	PAAD	184	179	183	177	4
**Cholangiocarcinoma**	CHOL	36	36	36	36	9
**Lung adenocarcinoma**	LUAD	566	566	511	510	59
**Prostate adenocarcinoma**	PRAD	494	494	489	493	52
**Glioblastoma multiforme**	GBM	592	397	575	160	5
**Brain Lower Grade Glioma**	LGG	514	512	511	514	
**Pheochromocytoma and Paraganglioma**	PCPG	178	178	161	178	3
**Skin Cutaneous Melanoma**	SKCM	448	440	367	443	1
**Uveal Melanoma**	UVM	80	80	80	80	
**Kidney renal clear cell carcinoma**	KIRC	512	402	509	510	72
**Kidney renal papillary cell carcinoma**	KIRP	283	276	283	283	32
**Kidney Chromophobe**	KICH	65	65	65	65	25
**Sarcoma**	SARC	255	254	253	253	2
**Liver hepatocellular carcinoma**	LIHC	372	366	367	366	50
**Adrenocortical carcinoma**	ACC	92	91	89	78	
**Mesothelioma**	MESO	87	86	87	87	
**Testicular Germ Cell Tumors**	TGCT	149	145	149	149	
**Thyroid carcinoma**	THCA	500	490	497	498	59
**Thymoma**	THYM	123	123	123	119	2
**Lymphoid Neoplasm Diffuse Large B-cell Lymphoma**	DLBC	48	41	48	48	
**Acute Myeloid Leukemia**	LAML	200	200	191	173	
		10967	10436	10712	10071	737

Table S3. Frequency (%) of METTL genetic amplifications in 32 tumor types from TCGA Pan-Cancer database

METTL Gene	Pan-Cancer	BRCA	OV	UCEC	UCS	CESC	BLCA	HNSC	LUSC	ESCA	STAD	COADREAD	PAAD	CHOL	LUAD	PRAD	GBM	LGG	PCPG	SKCM	UVM	KIRC	KIRP	KICH	SARC	LIHC	ACC	MESO	TGCT	THCA	THYM	DLBC	LAML
METTL1	2.29	1.31	0.87	0.19	0.00	0.00	1.23	0.58	0.41	0.55	2.28	0.17	1.64	5.56	5.29	0.20	13.57	4.12	1.24	3.54	0.00	0.00	0.00	0.20	17.00	1.36	6.74	0.00	0.00	0.00	0.00	2.08	0.00
METTL2A	1.73	6.73	2.45	0.57	0.00	1.02	2.21	0.00	0.82	2.75	2.51	0.84	2.19	0.00	1.57	0.61	0.00	1.37	1.24	1.91	1.25	0.00	0.71	0.20	1.19	3.00	2.25	5.75	0.00	0.20	1.63	0.00	0.00
METTL2B	0.97	0.93	4.37	0.96	1.79	0.34	0.98	0.19	0.62	0.00	0.46	0.17	0.00	0.00	1.18	0.61	2.26	1.57	0.00	3.00	0.00	1.54	1.06	0.39	0.79	0.54	0.00	0.00	0.00	0.00	0.00	0.00	0.00
METTL3	0.72	0.65	2.62	0.57	0.00	1.02	1.23	1.16	1.03	1.10	0.91	0.00	0.55	0.00	1.76	0.20	0.00	1.37	0.62	0.27	0.00	0.00	0.00	0.00	1.58	0.54	1.12	0.00	0.00	0.00	0.00	0.00	0.00
METTL4	1.16	0.37	2.45	2.29	3.57	1.02	3.92	3.87	2.67	4.40	0.68	0.17	3.28	0.00	1.37	0.41	0.00	0.39	0.00	0.54	0.00	1.54	0.00	0.00	0.79	1.09	0.00	0.00	0.00	0.20	0.00	0.00	0.52
METTL5	0.41	0.19	2.27	0.19	1.79	0.34	0.74	0.97	0.62	0.55	0.68	0.00	0.55	0.00	0.78	0.00	0.17	0.00	0.00	0.00	0.00	0.00	0.35	0.20	0.79	0.27	0.00	0.00	0.00	0.00	0.00	0.00	0.00
METTL6	0.52	0.84	1.22	0.38	0.00	0.00	4.66	0.00	0.00	0.55	0.68	0.17	0.00	0.00	0.20	0.00	0.35	0.59	0.62	0.27	0.00	0.00	0.00	0.00	1.98	0.27	0.00	0.00	0.00	0.00	0.00	0.00	0.00
METTL7A	0.54	0.28	0.70	0.76	5.36	0.68	1.72	0.00	0.21	1.65	0.00	0.17	0.00	0.00	0.39	0.82	0.35	0.59	0.00	0.54	0.00	0.00	0.00	0.00	3.16	0.54	5.62	0.00	0.67	0.00	0.00	2.08	0.00
METTL7B	0.54	0.00	1.05	1.15	1.79	0.00	0.98	0.19	0.21	0.00	1.37	0.17	1.64	0.00	1.18	1.02	0.70	0.20	0.62	0.82	0.00	0.00	0.00	0.00	1.98	0.27	2.25	0.00	0.00	0.00	0.00	2.08	0.00
METTL8	0.56	0.37	2.80	0.19	1.79	0.34	0.49	1.16	1.85	1.65	0.46	0.00	0.55	0.00	0.78	0.00	0.35	0.20	0.00	0.00	0.00	0.00	0.35	0.39	0.79	0.54	0.00	0.00	0.00	0.00	0.00	0.00	0.00
METTL9	0.64	3.27	1.05	0.57	0.00	0.00	2.70	0.00	0.00	0.55	0.00	0.34	0.00	0.00	0.20	1.02	0.00	0.20	0.00	0.00	0.00	0.00	0.35	0.00	0.40	0.27	0.00	0.00	0.00	0.00	0.00	2.08	0.00
EEF1AKMT2	0.57	1.03	2.80	1.15	1.79	0.00	0.25	0.00	0.41	1.10	2.51	0.17	0.55	0.00	0.39	0.41	0.00	0.20	1.24	0.00	0.00	0.00	0.00	0.00	0.00	0.00	2.25	0.00	0.00	0.00	0.00	0.00	0.00
NTMT1	0.49	0.28	1.75	1.15	1.79	0.00	0.25	0.77	0.21	1.10	1.14	0.00	0.00	0.00	0.00	0.61	0.52	1.18	0.00	0.00	0.00	1.54	0.35	0.00	0.79	0.54	1.12	0.00	0.00	0.20	0.00	0.00	0.00
METTL11B	2.46	6.82	1.92	2.10	0.00	1.02	7.11	0.39	3.90	2.75	1.83	0.68	3.28	11.11	4.31	0.20	0.17	0.00	3.11	1.91	0.00	0.00	0.00	0.20	6.32	8.17	0.00	1.15	0.00	0.00	1.63	4.17	0.00
CSKMT	0.41	0.65	0.87	1.15	1.79	0.00	1.47	0.77	0.00	1.10	0.23	0.00	0.55	2.78	0.59	0.00	0.00	0.39	0.62	0.00	0.00	0.00	0.00	0.00	0.00	0.27	2.25	1.15	0.00	0.00	0.00	0.00	0.00
EEF1AKNMT	2.51	6.64	2.62	2.68	3.57	1.02	7.11	0.39	3.70	2.20	1.83	0.68	2.73	11.11	3.92	0.00	0.17	0.20	3.11	1.63	0.00	0.00	0.00	0.20	8.30	8.17	1.12	1.15	0.00	0.00	1.63	2.08	0.00
METTL14	0.20	0.47	0.35	0.00	0.00	0.00	0.74	0.00	0.00	0.00	0.23	0.00	0.00	0.00	0.00	0.00	0.52	0.20	0.00	0.27	0.00	0.00	0.00	0.00	1.58	0.00	0.00	0.00	0.00	0.20	0.00	0.00	0.00
METTL15	0.42	0.47	1.40	0.38	0.00	0.00	1.23	0.19	0.62	1.10	1.60	0.17	0.55	0.00	0.39	0.41	0.17	0.20	0.62	0.00	0.00	0.00	0.00	0.00	1.19	0.00	0.00	0.00	0.00	0.00	0.00	0.00	0.00
METTL16	0.28	0.09	1.57	0.76	1.79	0.00	0.00	0.00	0.00	0.55	0.46	0.34	0.00	0.00	0.00	0.20	0.17	0.39	0.00	0.82	0.00	0.00	0.00	0.00	1.19	0.00	0.00	0.00	0.00	0.00	0.00	0.00	0.00
METTL17	0.86	0.47	3.67	1.34	0.00	1.02	1.72	1.35	1.44	1.10	0.91	0.00	0.55	0.00	1.76	0.41	0.17	1.18	0.62	0.27	0.00	0.00	0.00	0.00	1.19	0.82	1.12	0.00	0.00	0.00	0.00	0.00	0.52
METTL18	2.43	6.82	1.75	2.10	0.00	1.02	7.11	0.39	3.90	3.30	1.60	0.68	3.28	11.11	4.31	0.41	0.00	0.00	3.11	1.91	0.00	0.00	0.00	0.20	5.14	8.45	0.00	1.15	0.00	0.00	1.63	4.17	0.00
TRMT44	0.38	0.09	2.97	0.38	0.00	0.00	0.74	0.00	0.21	0.55	0.46	0.17	0.55	0.00	0.78	0.41	0.00	0.20	0.00	0.27	0.00	0.00	0.00	0.00	0.79	0.00	0.00	0.00	0.00	0.20	0.81	0.00	0.00
ETFBKMT	1.58	1.50	7.17	0.76	3.57	0.68	1.72	1.74	3.29	3.30	2.05	0.34	4.92	0.00	2.35	0.82	1.04	1.18	0.62	1.09	0.00	0.00	0.00	0.00	1.58	0.27	2.25	1.15	3.36	0.00	0.00	0.00	0.00
METTL21A	0.43	0.75	1.22	0.38	0.00	0.00	0.25	0.39	0.62	2.20	1.14	0.00	1.64	0.00	0.98	0.20	0.00	0.00	0.00	0.00	0.00	0.00	0.00	0.39	0.40	0.54	0.00	0.00	0.00	0.00	0.00	0.00	0.00
EEF1AKMT3	2.27	1.21	0.87	0.19	0.00	0.00	1.23	0.58	0.41	1.10	2.28	0.17	1.64	5.56	5.29	0.20	13.74	3.92	1.24	3.54	0.00	0.00	0.00	0.00	16.60	1.36	6.74	0.00	0.00	0.00	0.00	2.08	0.00
METTL21C	0.90	1.59	1.92	0.38	5.36	0.34	1.96	0.77	0.41	0.55	1.83	1.52	0.55	0.00	0.59	0.41	0.35	0.78	0.62	0.82	0.00	0.00	0.00	0.39	1.19	2.18	0.00	1.15	0.00	0.00	0.00	0.00	0.00
VCPKMT	0.58	1.12	1.22	0.38	0.00	0.68	0.00	0.97	0.21	1.10	1.14	0.17	0.00	0.00	2.16	0.82	0.00	0.39	0.62	0.27	0.00	0.00	0.00	0.00	1.19	0.54	0.00	0.00	0.00	0.00	0.00	2.08	0.00
METTL22	0.98	3.83	1.40	0.19	3.57	1.02	4.17	0.77	0.62	1.10	0.68	0.00	0.00	0.00	0.78	1.23	0.35	0.39	0.00	0.27	0.00	0.00	0.35	0.00	1.19	0.00	1.12	0.00	0.00	0.00	0.00	2.08	0.00
METTL23	1.45	3.83	2.62	1.53	1.79	1.71	1.72	0.19	1.85	1.65	0.46	0.51	0.55	0.00	1.76	0.41	0.17	1.76	1.24	3.00	1.25	0.00	0.00	0.20	1.98	3.54	0.00	3.45	0.00	0.20	0.81	0.00	0.00
METTL24	0.43	1.59	0.35	1.15	1.79	0.34	0.25	0.19	0.62	0.00	0.46	0.00	0.00	0.00	0.39	0.20	0.17	0.00	0.00	0.00	0.00	0.00	0.00	0.20	2.37	0.00	1.12	0.00	0.00	0.00	0.00	0.00	0.00
METTL25	0.48	0.75	0.35	0.00	0.00	0.00	0.98	0.00	0.00	1.10	0.46	0.00	0.00	0.00	0.59	0.82	0.35	0.00	0.62	0.82	0.00	0.00	0.00	0.00	5.93	0.27	3.37	1.15	0.00	0.00	0.00	0.00	0.00
RRNAD1	2.89	8.13	3.67	3.82	3.57	1.71	3.68	0.39	3.29	2.75	1.60	0.51	3.83	11.11	6.86	0.82	0.70	1.18	3.11	1.91	0.00	0.00	0.00	0.20	3.56	10.63	2.25	1.15	0.00	0.00	1.63	2.08	0.00
METTL26	0.78	3.74	1.75	0.96	0.00	0.34	0.25	0.19	0.00	0.00	0.91	0.17	0.00	0.00	0.59	1.02	0.35	0.20	0.00	0.00	0.00	1.54	1.06	0.20	0.79	0.00	1.12	0.00	0.00	0.20	0.00	2.08	0.00
METTL27	0.87	0.56	2.27	0.76	1.79	0.68	0.74	0.77	1.23	2.20	2.05	0.34	1.09	0.00	0.78	1.02	1.57	0.39	0.00	0.54	0.00	0.00	0.35	0.39	0.79	1.09	2.25	0.00	2.01	0.00	0.00	2.08	0.00

Note: METTLs that have higher frequencies of amplification in Pan-Cancer (>2%) or individual tumor type (>5%) are highlighted in color.

Table S4. Frequency (%) of METTL deep deletions in 32 tumor types from TCGA Pan-Cancer database

METTL Gene	Pan-Cancer	BRCA	OV	UCEC	UCS	CESC	BLCA	HNSC	LUSC	ESCA	STAD	COADREAD	PAAD	CHOL	LUAD	PRAD	GBM	LGG	PCPG	SKCM	UVM	KIRC	KIRP	KICH	SARC	LIHC	ACC	MESO	TGCT	THCA	THYM	DLBC	LAML
METTL1	0.01	0.00	0.00	0.00	0.00	0.00	0.00	0.00	0.00	0.00	0.00	0.00	0.00	0.00	0.00	0.00	0.00	0.20	0.00	0.00	0.00	0.00	0.00	0.00	0.00	0.00	0.00	0.00	0.00	0.00	0.00	0.00	0.00
METTL2A	0.04	0.00	0.17	0.00	0.00	0.34	0.00	0.00	0.00	0.00	0.00	0.00	0.00	0.00	0.00	0.20	0.00	0.00	0.00	0.00	0.00	0.00	0.00	0.00	0.00	0.27	0.00	0.00	0.00	0.00	0.00	0.00	0.00
METTL2B	0.13	0.09	0.00	0.00	0.00	0.00	0.25	0.19	0.41	1.10	0.23	0.00	0.00	0.00	0.00	0.41	0.00	0.00	0.00	0.00	0.00	0.00	0.00	0.00	0.40	0.00	0.00	0.00	0.00	0.00	0.00	0.00	1.57
METTL3	0.15	0.19	0.17	0.00	0.00	0.00	0.49	0.39	0.21	0.00	0.91	0.00	0.00	0.00	0.00	0.41	0.00	0.00	0.00	0.00	0.00	0.00	0.71	0.00	0.00	0.00	0.00	0.00	0.00	0.00	0.00	0.00	0.00
METTL4	0.17	0.47	0.17	0.00	0.00	0.00	0.00	0.00	0.00	0.55	0.00	0.17	0.00	0.00	0.39	0.20	0.35	0.00	0.62	0.00	0.00	0.00	0.35	0.00	0.00	0.00	0.00	0.00	1.34	0.00	0.00	2.08	0.00
METTL5	0.18	0.00	0.00	0.00	0.00	0.00	0.00	0.00	0.21	0.00	0.23	0.00	0.55	0.00	0.00	1.64	0.17	0.00	0.00	0.00	0.00	0.00	0.00	0.00	0.79	0.54	0.00	0.00	0.67	0.00	0.81	0.00	0.52
METTL6	0.26	0.00	0.35	0.19	0.00	0.00	0.00	0.39	0.82	0.55	0.00	0.00	0.00	0.00	0.00	0.61	0.00	0.00	0.00	0.27	0.00	2.55	0.00	0.00	0.00	0.00	0.00	0.00	0.00	0.00	0.81	0.00	0.00
METTL7A	0.09	0.00	0.00	0.00	0.00	0.00	0.00	0.00	0.00	0.00	0.46	0.00	0.55	0.00	0.00	0.41	0.17	0.59	0.00	0.00	0.00	0.00	0.00	0.00	0.40	0.00	0.00	0.00	0.00	0.00	0.00	0.00	0.00
METTL7B	0.06	0.00	0.00	0.00	0.00	0.00	0.25	0.00	0.00	0.00	0.23	0.00	0.00	0.00	0.00	0.00	0.17	0.59	0.00	0.00	0.00	0.00	0.00	0.00	0.00	0.00	0.00	0.00	0.00	0.00	0.00	0.00	0.00
METTL8	0.16	0.00	0.17	0.00	0.00	0.00	0.25	0.00	0.21	0.00	0.23	0.00	0.00	0.00	0.00	1.23	0.17	0.00	0.00	0.00	0.00	0.00	0.00	0.00	0.79	0.54	0.00	0.00	0.67	0.00	0.81	0.00	0.00
METTL9	0.08	0.00	0.17	0.19	0.00	0.00	0.00	0.19	0.00	0.00	0.00	0.17	0.00	0.00	0.39	0.00	0.35	0.00	0.00	0.27	0.00	0.00	0.00	0.00	0.00	0.00	0.00	0.00	0.00	0.00	0.00	0.00	0.00
EEF1AKMT2	0.32	0.09	0.00	0.00	0.00	0.00	1.47	0.00	0.41	0.55	0.00	0.00	0.00	0.00	0.20	0.82	0.35	1.96	0.00	1.09	0.00	0.20	0.00	0.00	0.40	0.00	0.00	1.15	0.00	0.00	0.00	0.00	0.00
NTMT1	0.21	0.37	0.70	0.19	0.00	0.00	0.49	0.00	0.41	0.00	0.23	0.00	0.55	0.00	0.20	0.00	0.00	0.00	0.00	0.00	0.00	0.20	0.00	0.00	0.00	0.00	0.00	0.00	0.67	0.82	0.81	0.00	0.00
METTL11B	0.06	0.00	0.00	0.00	0.00	0.00	0.00	0.00	0.00	0.00	0.00	0.00	0.00	0.00	0.20	0.61	0.00	0.00	0.00	0.00	0.00	0.00	0.35	0.00	0.40	0.00	0.00	0.00	0.00	0.00	0.00	0.00	0.00
CSKMT	0.09	0.00	0.17	0.19	1.79	0.00	0.00	0.00	0.00	0.00	0.23	0.34	0.00	0.00	0.00	0.41	0.00	0.00	0.00	0.00	1.25	0.00	0.00	0.00	0.00	0.27	0.00	0.00	0.00	0.00	0.00	0.00	0.00
EEF1AKNMT	0.05	0.00	0.00	0.00	0.00	0.00	0.00	0.00	0.00	0.00	0.00	0.00	0.00	0.00	0.20	0.41	0.00	0.00	0.00	0.00	0.00	0.00	0.35	0.00	0.40	0.00	0.00	0.00	0.00	0.00	0.00	0.00	0.00
METTL14	0.24	0.00	0.70	0.38	0.00	0.00	0.00	0.00	0.00	0.00	0.46	0.17	0.00	0.00	0.00	2.25	0.00	0.39	0.00	0.00	0.00	0.39	0.00	0.00	0.79	0.00	0.00	0.00	0.00	0.00	0.00	0.00	0.00
METTL15	0.11	0.09	0.00	0.00	0.00	0.00	0.25	0.00	0.00	0.00	0.46	0.51	0.00	0.00	0.00	0.00	0.00	0.59	0.00	0.00	0.00	0.00	0.00	0.00	0.40	0.00	1.12	0.00	0.00	0.00	0.00	0.00	0.00
METTL16	0.53	0.47	0.17	0.00	0.00	1.02	0.98	0.39	0.41	1.10	0.91	1.18	0.00	0.00	1.37	0.41	0.00	0.00	0.62	0.27	0.00	0.00	0.35	0.00	0.00	2.18	2.25	0.00	0.00	0.41	0.81	2.08	0.52
METTL17	0.16	0.09	0.17	0.00	0.00	0.00	0.74	0.19	0.21	0.00	0.91	0.00	0.00	0.00	0.00	0.41	0.00	0.00	0.00	0.00	0.00	0.00	1.41	0.00	0.00	0.00	0.00	0.00	0.00	0.00	0.00	0.00	0.00
METTL18	0.06	0.00	0.00	0.00	0.00	0.00	0.00	0.00	0.00	0.00	0.00	0.00	0.00	0.00	0.20	0.61	0.00	0.00	0.00	0.00	0.00	0.00	0.35	0.00	0.40	0.00	0.00	0.00	0.00	0.00	0.00	0.00	0.00
TRMT44	0.31	0.56	0.35	0.38	0.00	1.37	0.25	0.58	0.62	1.10	0.46	0.34	0.00	0.00	0.39	0.00	0.17	0.00	0.00	0.27	0.00	0.00	0.00	0.00	0.79	0.00	0.00	0.00	0.00	0.00	0.00	0.00	0.00
ETFBKMT	0.10	0.09	0.00	0.00	0.00	0.00	0.00	0.19	0.00	0.55	0.46	0.17	0.00	0.00	0.39	0.00	0.17	0.20	0.00	0.00	0.00	0.00	0.00	0.00	0.40	0.00	0.00	0.00	0.00	0.00	0.00	0.00	0.00
METTL21A	0.29	0.37	0.00	0.38	0.00	2.05	0.74	1.16	0.21	0.55	0.23	0.00	0.00	0.00	0.00	0.00	0.17	0.00	0.00	0.00	1.25	0.20	0.71	0.00	0.79	0.00	0.00	0.00	0.00	0.00	0.00	0.00	0.00
EEF1AKMT3	0.01	0.00	0.00	0.00	0.00	0.00	0.00	0.00	0.00	0.00	0.00	0.00	0.00	0.00	0.00	0.00	0.00	0.20	0.00	0.00	0.00	0.00	0.00	0.00	0.00	0.00	0.00	0.00	0.00	0.00	0.00	0.00	0.00
METTL21C	0.24	0.37	0.52	0.00	0.00	0.00	0.00	0.19	0.21	0.55	0.46	0.17	0.00	0.00	0.59	1.02	0.17	0.00	0.00	0.27	0.00	0.00	0.00	0.00	0.79	0.00	0.00	0.00	0.00	0.00	0.00	2.08	0.00
VCPKMT	0.05	0.00	0.00	0.00	0.00	0.34	0.00	0.00	0.41	0.00	0.23	0.00	0.00	0.00	0.00	0.00	0.00	0.00	0.00	0.00	0.00	0.00	0.35	0.00	0.00	0.00	0.00	0.00	0.00	0.00	0.00	0.00	0.00
METTL22	0.07	0.00	0.35	0.00	0.00	0.00	0.49	0.00	0.21	0.00	0.00	0.17	0.00	0.00	0.20	0.00	0.00	0.00	0.00	0.00	0.00	0.00	0.00	0.00	0.00	0.27	0.00	0.00	0.00	0.00	0.00	0.00	0.00
METTL23	0.09	0.19	0.00	0.00	0.00	0.68	0.00	0.19	0.21	0.00	0.23	0.00	0.00	0.00	0.00	0.20	0.00	0.00	0.00	0.27	0.00	0.00	0.00	0.00	0.40	0.00	0.00	0.00	0.00	0.00	0.00	0.00	0.00
METTL24	0.89	0.56	0.87	0.00	0.00	0.34	1.47	0.39	0.00	0.55	1.14	0.17	0.55	0.00	0.39	8.38	0.00	0.00	0.62	1.36	6.25	0.20	0.00	0.00	0.00	1.91	0.00	0.00	0.00	0.00	0.00	10.42	0.00
METTL25	0.08	0.09	0.17	0.00	0.00	0.00	0.00	0.19	0.00	0.00	0.46	0.00	0.00	0.00	0.20	0.00	0.17	0.20	0.00	0.00	0.00	0.00	0.00	0.00	0.00	0.27	0.00	0.00	0.00	0.00	0.00	0.00	0.00
RRNAD1	0.06	0.00	0.00	0.00	0.00	0.00	0.25	0.00	0.00	0.00	0.00	0.00	0.00	0.00	0.00	0.61	0.00	0.00	0.00	0.27	0.00	0.00	0.35	0.00	0.00	0.00	0.00	0.00	0.00	0.00	0.00	0.00	0.00
METTL26	0.53	0.19	1.22	0.57	3.57	0.34	1.47	0.00	0.41	2.75	1.37	0.51	0.55	0.00	0.39	0.61	0.00	0.20	0.00	0.54	0.00	0.00	0.71	0.00	0.40	0.82	0.00	0.00	0.67	0.20	0.00	4.17	0.52
METTL27	0.09	0.00	0.35	0.00	0.00	0.00	0.00	0.00	0.21	0.00	0.46	0.00	0.00	0.00	0.39	0.00	0.00	0.20	0.00	0.00	0.00	0.00	0.00	0.00	0.40	0.00	0.00	0.00	0.00	0.20	0.00	0.00	0.00

Note: METTLs that have higher frequencies of deep deletion in individual tumor type (>5%) are highlighted in color.

Table S5. Frequency (%) of METTL mutations in 32 tumor types from TCGA Pan-Cancer database

METTL Gene	Pan-Cancer	BRCA	OV	UCEC	UCS	CESC	BLCA	HNSC	LUSC	ESCA	STAD	COADREAD	PAAD	CHOL	LUAD	PRAD	GBM	LGG	PCPG	SKCM	UVM	KIRC	KIRP	KICH	SARC	LIHC	ACC	MESO	TGCT	THCA	THYM	DLBC	LAML
METTL1	0.48	0.19	0.00	1.91	0.00	0.34	0.00	0.77	0.00	0.00	0.46	0.84	0.00	0.00	0.78	0.20	0.70	0.00	0.62	2.45	0.00	0.00	0.35	0.00	1.58	0.54	0.00	0.00	0.00	0.00	0.00	2.08	0.00
METTL2A	0.53	0.37	0.35	3.44	0.00	0.34	1.47	0.00	0.21	0.00	0.91	1.01	0.55	0.00	0.78	0.00	0.17	0.20	0.00	1.36	0.00	0.00	0.00	0.00	0.00	0.27	0.00	0.00	0.00	0.00	0.00	4.17	0.00
METTL2B	0.60	0.47	0.17	4.02	0.00	0.34	0.98	0.58	0.21	0.00	0.00	1.18	0.55	0.00	0.78	0.00	0.17	0.20	0.00	2.45	0.00	0.00	0.35	0.00	0.00	1.09	0.00	0.00	0.00	0.00	0.00	0.00	0.00
METTL3	0.86	0.28	0.35	3.44	0.00	1.71	4.41	0.97	0.41	0.55	0.23	1.35	0.55	0.00	0.78	0.41	0.17	0.78	0.00	2.72	0.00	0.59	0.71	0.00	0.00	0.00	0.00	0.00	0.00	0.20	0.00	0.00	0.52
METTL4	0.78	0.19	0.00	4.21	0.00	0.34	0.98	0.00	1.64	1.10	1.37	0.51	0.55	0.00	0.98	0.41	0.35	0.00	0.00	4.63	0.00	0.20	0.71	0.00	0.40	0.27	0.00	0.00	0.67	0.00	0.00	6.25	0.00
METTL5	0.29	0.09	0.00	2.49	0.00	0.34	0.74	0.19	0.21	0.00	0.46	0.34	0.00	0.00	0.20	0.00	0.35	0.00	0.00	0.54	0.00	0.00	0.35	0.00	0.00	0.27	0.00	0.00	0.00	0.00	0.00	0.00	0.00
METTL6	0.49	0.19	0.00	2.68	0.00	0.68	1.23	0.39	0.41	0.00	1.14	1.69	0.55	0.00	0.20	0.20	0.17	0.00	0.00	0.82	0.00	0.00	0.35	0.00	0.00	0.54	0.00	0.00	0.00	0.00	0.00	0.00	0.52
METTL7A	0.35	0.19	0.17	1.15	0.00	0.68	0.74	0.00	0.21	0.00	0.46	0.84	0.00	0.00	0.00	0.20	0.35	0.00	0.00	2.45	0.00	0.00	0.35	0.00	0.79	0.27	0.00	0.00	0.00	0.00	0.00	0.00	0.00
METTL7B	0.36	0.28	0.00	1.34	0.00	0.00	0.00	0.19	0.62	0.00	0.23	0.84	0.55	0.00	0.59	0.00	0.00	0.00	0.00	3.54	0.00	0.20	0.00	0.00	0.00	0.00	1.12	0.00	0.00	0.00	0.00	0.00	0.00
METTL8	0.47	0.19	0.52	1.72	0.00	1.02	0.74	0.77	0.62	0.00	0.68	0.51	0.00	2.78	0.20	0.00	0.17	0.00	0.00	2.72	0.00	0.00	0.35	0.00	0.40	0.00	0.00	0.00	0.00	0.00	0.00	0.00	1.05
METTL9	0.37	0.28	0.17	1.53	0.00	0.34	0.00	0.19	0.62	0.55	0.68	0.51	0.00	0.00	0.39	0.41	0.00	0.00	0.00	1.91	0.00	0.00	0.71	0.00	0.40	0.27	0.00	0.00	0.00	0.00	0.00	0.00	0.52
EEF1AKMT2	0.30	0.19	0.00	1.91	0.00	0.00	0.00	0.19	0.21	1.10	0.23	0.51	0.00	0.00	0.59	0.41	0.00	0.00	0.00	1.63	0.00	0.00	0.35	0.00	0.00	0.00	0.00	0.00	0.00	0.00	0.00	0.00	0.00
NTMT1	0.34	0.19	0.00	2.10	0.00	0.68	0.25	0.19	0.21	0.00	1.37	0.68	1.09	0.00	0.20	0.20	0.35	0.00	0.00	0.27	0.00	0.00	0.00	0.00	0.40	0.00	0.00	0.00	0.00	0.00	0.00	0.00	0.00
METTL11B	0.33	0.47	0.00	3.63	0.00	0.00	0.00	0.00	0.00	0.55	0.00	1.35	0.00	0.00	0.00	0.00	0.00	0.00	0.00	0.00	0.00	0.00	0.00	0.00	0.40	0.27	0.00	0.00	0.00	0.00	0.00	0.00	0.00
CSKMT	0.32	0.09	0.17	1.72	0.00	0.34	1.47	0.19	0.41	0.00	1.14	0.17	0.00	0.00	0.39	0.00	0.00	0.00	0.00	1.36	0.00	0.00	0.00	0.00	0.00	0.00	0.00	0.00	0.00	0.00	0.00	0.00	0.00
EEF1AKNMT	1.09	0.28	0.17	4.59	0.00	1.37	1.72	1.16	2.67	1.10	1.37	2.53	0.55	0.00	1.76	0.61	0.35	0.00	0.00	3.00	0.00	0.39	0.35	0.00	1.98	0.54	0.00	0.00	0.00	0.00	0.00	0.00	0.00
METTL14	0.73	0.37	0.17	4.21	1.79	0.68	1.47	0.58	0.62	0.00	0.68	1.01	0.55	0.00	1.37	0.41	0.35	0.39	0.62	1.36	0.00	0.20	0.71	0.00	0.00	0.00	1.12	0.00	0.00	0.00	1.63	0.00	0.52
METTL15	0.61	0.19	0.52	4.21	0.00	0.34	0.25	0.39	0.00	1.10	1.14	1.35	1.09	0.00	0.78	0.20	0.35	0.00	0.00	1.09	0.00	0.00	0.35	1.54	1.19	0.27	0.00	0.00	0.00	0.00	0.00	0.00	0.00
METTL16	0.63	0.19	0.17	2.68	0.00	0.34	0.49	0.58	1.23	0.00	0.68	1.69	0.00	0.00	0.59	0.00	0.17	0.20	0.00	3.00	0.00	0.59	0.71	0.00	0.79	0.00	0.00	0.00	0.00	0.20	0.00	2.08	0.00
METTL17	0.75	0.37	0.17	4.21	1.79	0.68	0.74	0.77	1.03	0.55	2.28	0.34	0.55	0.00	1.17	0.41	0.00	0.00	0.00	3.00	0.00	0.20	0.35	0.00	0.00	0.54	0.00	0.00	0.00	0.00	0.81	0.00	0.00
METTL18	0.46	0.37	0.00	3.06	0.00	1.02	0.98	0.19	0.62	0.55	0.23	0.68	0.00	0.00	0.78	0.20	0.17	0.20	0.00	0.27	0.00	0.20	0.00	0.00	0.00	0.82	0.00	0.00	0.00	0.00	0.00	0.00	0.00
TRMT44	0.69	0.19	0.70	4.21	0.00	0.68	0.49	0.39	0.00	1.10	0.68	0.84	0.00	0.00	0.59	0.41	0.17	0.00	0.00	4.63	0.00	0.20	1.06	0.00	0.40	0.27	0.00	0.00	0.67	0.00	0.00	0.00	0.00
ETFBKMT	0.34	0.09	0.00	2.29	0.00	0.34	0.49	0.39	0.21	0.00	0.46	0.34	0.00	0.00	0.59	0.00	0.35	0.20	0.00	1.63	0.00	0.00	0.00	0.00	0.00	0.27	0.00	0.00	0.00	0.00	0.00	0.00	0.00
METTL21A	0.34	0.09	0.35	1.91	1.79	0.34	0.25	0.39	0.21	0.00	1.14	1.01	0.00	0.00	0.59	0.00	0.00	0.00	0.62	0.54	0.00	0.00	0.00	0.00	0.00	0.00	0.00	0.00	0.00	0.00	0.00	0.00	0.00
EEF1AKMT3	0.45	0.37	0.17	1.53	0.00	0.00	0.74	0.19	0.41	0.00	0.46	0.51	0.00	0.00	0.39	0.00	0.87	0.59	0.00	1.63	0.00	0.00	0.35	0.00	2.37	0.27	0.00	0.00	0.00	0.00	0.00	0.00	0.00
METTL21C	0.64	0.37	0.00	2.87	1.79	0.68	0.98	0.39	0.41	0.00	0.46	0.84	0.55	0.00	0.78	0.20	0.17	0.20	0.62	3.54	0.00	0.20	0.71	0.00	0.40	1.63	0.00	0.00	0.00	0.00	0.00	0.00	0.00
VCPKMT	0.25	0.00	0.17	1.53	0.00	0.00	0.49	0.00	0.62	0.00	0.23	0.84	0.00	0.00	0.39	0.00	0.00	0.00	0.00	1.09	0.00	0.00	0.00	0.00	0.00	0.00	0.00	0.00	0.00	0.00	0.00	2.08	0.00
METTL22	0.55	0.19	0.17	0.76	0.00	0.00	0.25	0.97	1.23	0.55	1.60	1.35	0.00	0.00	1.57	0.00	0.35	0.20	0.00	2.18	0.00	0.00	0.71	0.00	0.40	0.27	0.00	0.00	0.00	0.00	0.00	0.00	0.52
METTL23	0.29	0.56	0.00	0.19	0.00	0.34	1.23	0.39	0.62	0.00	0.23	0.68	0.00	0.00	0.39	0.20	0.00	0.00	0.00	0.82	0.00	0.00	0.35	0.00	0.40	0.00	0.00	0.00	0.00	0.00	0.00	0.00	0.00
METTL24	0.62	0.28	0.00	3.82	0.00	0.68	1.23	0.00	1.44	0.00	0.68	1.01	0.00	0.00	0.39	0.20	0.35	0.39	0.00	2.72	0.00	0.20	0.00	0.00	0.40	0.27	0.00	0.00	0.00	0.00	0.00	0.00	0.00
METTL25	0.93	0.47	0.17	3.44	0.00	1.02	1.96	0.39	1.64	1.10	1.37	1.86	0.55	0.00	1.76	0.20	0.35	0.20	0.00	2.18	0.25	0.20	0.35	0.00	3.16	0.27	1.12	0.00	0.00	0.20	0.00	0.00	0.00
RRNAD1	0.65	0.65	0.00	4.02	1.79	1.02	0.49	0.19	0.21	0.00	1.14	1.52	0.00	0.00	0.20	0.20	0.52	0.39	0.00	1.91	0.25	0.00	0.71	0.00	0.00	0.00	2.25	1.15	0.00	0.00	0.00	0.00	0.00
METTL26	0.12	0.09	0.17	0.38	0.00	0.00	0.00	0.00	0.00	0.00	0.00	0.17	0.00	0.00	0.20	0.00	0.00	0.20	0.00	0.54	0.00	0.00	1.06	0.00	0.00	0.27	0.00	0.00	0.00	0.00	0.00	0.00	0.00
METTL27	0.45	0.37	0.17	2.49	0.00	0.34	0.25	0.58	0.41	0.55	0.46	1.01	0.55	0.00	0.78	0.00	0.00	0.00	0.00	1.63	0.00	0.00	0.35	0.00	0.00	0.54	0.00	0.00	0.00	0.00	0.00	0.00	0.00

Note: METTL4 that has highest frequency of mutation in DLBC (>5%) is highlighted in color.

Table S6. mRNA expression difference (log2 FC) for METTLs between TCGA tumor and normal samples

METTL gene	BRCA	UCEC	BLCA	HNSC	LUSC	ESCA	STAD	COADREAD	LUAD	PRAD	KIRC	KIRP	KICH	LIHC	THCA
METTL1	0.686	0.415	0.934	0.349	1.459	1.036	0.876	1.404	1.291	0.558	0.249	0.189	0.262	0.556	-0.364
METTL2A	0.390	0.680	0.512	0.032	0.782	0.248	0.300	0.330	0.686	0.175	-0.178	0.364	-0.249	0.384	-0.185
METTL2B	0.096	0.311	0.184	-0.227	0.576	0.139	0.342	0.551	0.485	0.128	-0.126	0.189	0.133	0.355	-0.296
METTL3	-0.050	0.339	0.525	0.315	0.187	0.217	0.217	0.554	0.377	0.564	0.078	0.197	0.268	0.672	-0.191
METTL4	-0.097	-0.044	0.155	0.416	0.479	0.420	0.441	-0.126	0.218	-0.298	-0.130	-0.406	0.068	0.336	-0.574
METTL5	-0.037	0.555	0.236	0.322	1.045	0.271	0.102	0.676	0.732	0.095	0.354	0.265	-0.031	0.596	0.205
METTL6	0.268	0.268	0.632	-0.073	0.283	0.034	0.091	0.592	0.195	0.219	-0.260	0.346	0.195	0.770	-0.112
METTL7A	-1.891	-1.841	-0.934	-2.474	-2.188	-2.970	-1.753	-2.660	-1.993	-1.003	-0.824	-0.767	0.113	-0.962	-0.879
METTL7B	-0.616	3.314	2.562	0.383	-0.012	2.525	1.706	-0.454	2.080	-0.640	0.756	0.746	-2.738	-0.771	4.412
METTL8	-0.022	0.184	-0.203	0.232	1.388	0.941	0.385	0.507	0.461	0.060	-0.157	-0.029	-0.685	-0.145	-0.608
METTL9	-0.002	-0.074	-0.073	-0.357	0.493	-0.209	-0.083	0.340	0.406	0.224	0.024	0.602	-0.692	0.208	0.332
EEF1AKMT2	-0.170	-0.159	0.041	-0.153	0.272	-0.071	0.168	-0.111	0.044	0.240	-0.167	-0.501	-0.387	-0.262	-0.423
NTMT1	0.624	0.520	0.529	0.455	0.735	0.558	0.145	1.012	0.767	0.046	-0.277	-0.110	0.776	0.180	0.414
CSKMT	0.171	-0.093	0.818	-0.010	0.516	0.147	0.123	0.094	0.375	0.750	-0.123	-0.436	-0.569	0.694	0.302
EEF1AKNMT	0.522	0.389	0.512	0.116	0.477	0.541	0.504	0.259	0.488	0.026	-0.204	-0.057	-0.690	0.544	-0.161
METTL14	-0.363	-0.671	-0.596	-0.138	-0.477	-0.269	-0.168	-0.274	-0.232	-0.239	-0.233	-0.613	0.034	-0.538	-0.461
METTL15	0.121	-0.035	-0.341	-0.164	-0.066	0.053	0.312	-0.059	0.098	-0.121	-0.052	0.079	0.448	-0.037	-0.239
METTL16	-0.432	-0.618	-0.778	0.003	-0.418	-0.062	0.262	0.124	-0.282	-0.171	-0.018	0.231	0.013	0.118	-0.037
METTL17	-0.114	0.464	0.506	0.238	0.525	0.150	0.186	0.103	0.662	0.201	0.218	0.316	0.565	0.237	-0.106
METTL18	0.479	0.026	0.554	-0.029	0.359	0.291	0.298	0.103	0.648	0.231	-0.018	-0.441	-0.497	1.007	0.012
TRMT44	-0.296	-0.257	0.016	-0.512	-0.063	-0.070	-0.042	0.330	0.013	0.013	-0.061	-0.312	0.153	0.003	-0.060
ETFBKMT	-0.317	-0.518	-0.584	-0.375	-0.760	-1.339	-0.485	-1.142	-0.292	-0.062	-0.671	-0.575	-0.660	-1.005	-0.946
METTL21A	0.297	0.646	0.278	0.187	0.361	0.292	0.733	0.258	0.703	-0.006	-0.210	0.192	-0.697	0.377	-0.186
EEF1AKMT3	-0.539	-0.957	0.376	0.138	0.518	-0.035	0.123	0.715	0.674	0.233	0.552	-0.110	-0.473	0.078	-0.583
VCPKMT	-0.013	0.175	0.104	0.309	0.756	0.011	0.203	0.223	0.301	0.058	0.101	0.349	-0.479	-0.013	0.055
METTL22	-0.038	-0.245	-0.031	0.383	0.596	-0.293	-0.065	0.461	0.448	-0.108	0.639	0.624	0.279	-0.084	-0.214
METTL23	0.256	0.036	0.447	0.343	0.479	0.516	0.076	0.321	0.562	0.173	0.020	0.588	-0.540	0.404	-0.197
METTL24	-1.595	-1.286	-3.373	-1.626	-0.678	-1.915	-2.394	-3.481	-0.898	-1.307	-0.440	-1.456	-3.115	-0.086	-2.312
METTL25	-0.538	-0.341	-0.431	-0.610	-0.430	-0.092	-0.372	-0.656	-0.234	0.103	-0.199	-0.176	-0.659	0.251	-0.384
RRNAD1	0.399	0.112	0.317	-0.174	0.100	-0.065	-0.188	0.002	0.453	0.018	-0.168	-0.060	-0.148	0.392	-0.173
METTL26	1.088	0.597	0.934	0.453	0.437	0.367	0.092	0.917	0.744	0.782	1.330	0.771	-0.049	0.042	0.417
METTL27	0.967	-0.065	0.907	-0.216	0.000	2.810	1.562	0.730	0.405	0.365	0.998	1.840	-0.294	0.690	0.126

Table S7. mRNA expression difference (FDR) for METTLs between TCGA tumor and normal samples

METTL gene	BRCA	UCEC	BLCA	HNSC	LUSC	ESCA	STAD	LUAD	COADREAD	PRAD	KIRC	KICH	KIRP	LIHC	THCA
METTL1	6.99E-32	3.01E-04	1.86E-06	1.28E-04	5.01E-24	1.01E-04	6.45E-11	3.71E-28	1.68E-27	1.93E-11	3.36E-03	8.56E-02	1.60E-01	8.73E-07	3.91E-05
METTL2A	1.03E-24	3.85E-11	1.72E-05	4.31E-01	2.83E-20	1.34E-01	1.85E-05	8.62E-23	9.19E-07	7.69E-04	1.34E-06	1.11E-03	2.46E-06	5.91E-11	4.92E-05
METTL2B	4.01E-03	1.63E-03	1.24E-02	1.29E-02	9.35E-14	5.13E-01	3.00E-04	1.09E-17	5.55E-14	1.18E-01	1.48E-02	7.41E-01	5.26E-02	1.30E-06	4.11E-07
METTL3	3.59E-01	9.03E-04	5.86E-05	6.28E-05	7.06E-03	7.09E-02	1.28E-03	1.63E-07	1.61E-13	8.95E-17	2.26E-01	4.04E-03	1.03E-01	2.97E-17	1.16E-04
METTL4	3.32E-02	1.43E-01	2.09E-01	9.01E-06	1.69E-08	7.10E-03	8.54E-06	1.29E-03	8.14E-02	2.23E-09	3.81E-03	9.26E-01	1.41E-07	2.46E-05	2.98E-27
METTL5	3.59E-01	1.25E-12	5.80E-02	9.36E-05	1.00E-23	7.09E-02	7.77E-01	2.64E-18	1.69E-15	4.75E-02	6.00E-12	6.15E-01	3.73E-04	6.69E-12	7.12E-05
METTL6	6.52E-20	9.86E-07	4.33E-08	5.53E-01	1.30E-07	4.55E-01	2.13E-01	2.81E-06	5.23E-16	1.44E-06	7.01E-07	1.52E-01	6.84E-06	6.20E-24	6.10E-08
METTL7A	2.90E-61	9.95E-15	2.87E-03	2.65E-20	4.87E-27	1.03E-04	1.50E-16	1.47E-31	1.10E-30	4.06E-21	3.83E-21	5.17E-01	1.87E-09	1.73E-16	6.31E-17
METTL7B	3.12E-04	1.29E-07	3.75E-04	7.25E-01	9.06E-01	5.11E-02	3.37E-04	7.95E-19	6.13E-06	5.16E-03	1.78E-07	4.06E-08	1.47E-04	9.37E-08	1.57E-29
METTL8	4.57E-01	1.54E-01	2.81E-01	2.34E-02	6.81E-28	2.33E-02	2.60E-01	2.64E-15	3.47E-08	4.64E-01	1.31E-01	9.60E-05	5.96E-01	4.18E-01	3.90E-20
METTL9	6.14E-01	3.44E-01	5.64E-01	1.25E-09	4.18E-11	3.00E-01	1.76E-02	4.73E-09	6.05E-09	2.51E-04	7.47E-01	1.37E-07	3.12E-07	1.89E-02	1.03E-15
EEF1AKMT2	3.14E-04	9.87E-02	5.64E-01	1.40E-02	9.94E-04	9.71E-01	2.16E-02	7.04E-01	1.57E-01	2.51E-04	8.25E-05	4.70E-05	1.87E-09	1.97E-04	1.63E-18
NTMT1	2.02E-27	1.33E-07	9.81E-03	6.71E-06	1.06E-17	7.92E-04	1.76E-02	7.23E-20	1.30E-27	4.35E-01	5.71E-09	2.25E-05	1.32E-01	3.76E-01	9.00E-15
CSKMT	1.05E-01	7.81E-01	1.28E-04	7.38E-01	1.07E-07	1.82E-01	2.26E-01	3.79E-05	3.65E-01	1.25E-11	2.56E-02	6.11E-03	3.47E-04	7.22E-09	3.76E-09
EEF1AKNMT	2.20E-30	6.62E-06	3.37E-04	1.20E-03	2.92E-18	1.18E-03	2.57E-09	8.57E-21	2.04E-11	4.99E-01	8.03E-09	1.35E-08	2.67E-01	2.05E-14	4.05E-04
METTL14	1.98E-19	1.02E-12	4.44E-05	2.34E-02	9.17E-14	5.42E-02	1.26E-01	2.95E-09	3.17E-11	3.57E-05	1.20E-08	8.10E-01	2.66E-11	7.22E-09	8.38E-20
METTL15	1.06E-02	1.03E-01	3.45E-03	2.89E-02	3.06E-01	9.71E-01	1.45E-04	3.02E-02	9.53E-01	5.16E-03	3.93E-01	4.31E-05	3.77E-02	3.62E-01	2.57E-10
METTL16	1.65E-20	2.23E-11	1.86E-06	6.97E-01	1.33E-08	8.20E-01	2.39E-01	9.20E-07	3.19E-02	1.83E-05	7.47E-01	7.52E-01	2.70E-03	2.59E-01	1.48E-01
METTL17	2.65E-01	1.33E-07	5.20E-04	4.21E-02	1.70E-11	7.94E-02	1.54E-01	3.75E-14	2.11E-01	7.16E-02	2.51E-03	2.55E-07	2.77E-04	6.76E-04	2.75E-02
METTL18	3.91E-19	6.02E-01	3.37E-04	9.78E-01	7.52E-08	1.41E-01	1.23E-02	1.70E-19	1.18E-01	4.92E-04	8.59E-01	1.42E-04	5.46E-05	5.29E-17	5.40E-02
TRMT44	4.65E-12	9.03E-04	4.59E-01	5.47E-06	6.15E-01	4.55E-01	7.75E-01	3.05E-01	2.90E-10	5.28E-01	7.86E-02	2.62E-01	3.12E-07	6.03E-01	1.93E-01
ETFBKMT	1.15E-08	3.67E-06	1.59E-03	1.52E-04	7.70E-14	4.63E-03	3.95E-04	1.19E-05	2.03E-23	8.86E-01	8.43E-27	1.59E-07	8.54E-11	6.31E-15	2.16E-22
METTL21A	3.23E-15	3.85E-11	2.26E-02	2.47E-02	1.29E-08	4.55E-01	1.95E-07	1.15E-22	1.34E-07	7.34E-01	1.06E-04	7.57E-08	4.05E-04	7.08E-08	1.55E-07
EEF1AKMT3	1.65E-27	4.68E-14	1.36E-02	3.00E-01	1.68E-11	3.93E-01	2.15E-01	5.97E-21	4.04E-16	6.90E-03	4.31E-08	3.11E-05	1.43E-01	3.13E-01	9.29E-14
VCPKMT	9.72E-01	9.54E-02	6.07E-01	2.93E-04	6.86E-15	4.55E-01	2.19E-02	6.03E-07	5.96E-03	9.56E-01	5.82E-02	1.15E-05	5.26E-02	8.45E-01	9.68E-01
METTL22	4.04E-02	5.63E-03	9.40E-01	1.84E-02	2.45E-16	1.82E-01	2.81E-01	2.26E-14	9.63E-09	7.81E-02	3.85E-26	5.62E-04	7.49E-13	4.53E-01	3.91E-05
METTL23	7.67E-10	2.96E-01	3.37E-04	6.25E-05	5.98E-11	2.27E-02	2.26E-01	2.02E-20	2.77E-07	3.85E-04	9.05E-01	5.21E-07	3.87E-10	7.08E-08	4.12E-06
METTL24	9.70E-24	2.25E-04	3.16E-06	1.27E-06	2.64E-02	2.34E-02	2.51E-06	4.65E-08	1.54E-23	8.23E-11	3.81E-03	1.50E-08	2.48E-04	7.87E-01	2.26E-17
METTL25	1.32E-23	2.10E-04	1.03E-03	4.56E-13	1.69E-08	5.00E-01	8.66E-04	9.26E-04	4.24E-13	6.30E-02	8.55E-06	1.83E-08	1.64E-02	9.40E-03	4.77E-17
RRNAD1	2.16E-12	3.44E-01	2.14E-02	1.13E-02	3.50E-02	9.71E-01	1.47E-01	8.65E-14	8.00E-01	2.78E-01	4.23E-04	2.64E-01	8.90E-01	1.64E-05	1.77E-04
METTL26	8.10E-38	5.99E-06	5.06E-06	3.67E-05	2.70E-09	2.85E-01	9.55E-01	7.16E-16	5.07E-22	7.58E-12	1.98E-29	7.78E-01	8.62E-09	8.31E-01	1.13E-06
METTL27	3.08E-08	3.44E-01	7.15E-03	4.31E-01	8.53E-01	6.07E-04	9.65E-06	9.26E-04	1.99E-04	1.44E-01	2.91E-08	4.92E-01	2.42E-11	1.07E-03	6.36E-01

Table S8. Protein expression difference (log2 FC and FDR) for METTLs between CPTAC tumor and normal samples

METTL gene	BRCA (FC)	BRCA (FDR)	UCEC (FC)	UCEC (FDR)	COAD (FC)	COAD (FDR)	ccRCC (FC)	ccRCC (FDR)	LUAD (FC)	LUAD (FDR)	OV (FC)	OV (FDR)
METTL1	0.38	2.13E-02	0.39	1.25E-09	0.58	1.51E-18	0.20	1.17E-10	1.54	2.71E-18		
METTL2A	1.08	8.34E-05	0.74	4.86E-04	0.65	2.27E-02						
METTL2B	-0.65	3.22E-04	0.42	2.22E-09	0.19	2.53E-02	0.29	4.92E-15	1.62	1.15E-22		
METTL3	0.04	9.88E-02	-0.17	3.91E-05	-0.01	4.24E-01	0.04	2.18E-01	-0.07	2.78E-01	0.36	6.25E-07
METTL5	-0.34	4.11E-03	0.21	1.32E-06	-0.10	7.06E-01	0.47	2.03E-25	0.15	8.24E-01		
METTL7A	-1.15	8.02E-08	-0.51	7.69E-08	-1.27	1.06E-30	-0.56	1.05E-08	-3.11	3.95E-29		
METTL7B			0.83	2.05E-06	-0.09	8.87E-02	-0.03	7.25E-01	2.30	1.26E-15	-0.45	5.62E-02
METTL8			0.15	3.89E-01			-0.78	1.01E-08				
METTL9			0.17	2.25E-02			0.08	8.25E-02	0.23	3.81E-01		
EEF1AKMT2	-0.28	1.53E-02	0.08	3.34E-02			0.07	1.59E-01	-0.05	3.95E-01		
NTMT1	0.31	2.78E-05	0.06	2.62E-01	0.38	5.76E-10	-0.05	2.25E-01	0.93	5.91E-14	0.22	5.04E-01
EEF1AKNMT			0.19	2.51E-06	0.14	1.21E-05	0.05	6.79E-03	0.23	5.40E-03	-0.43	5.05E-05
METTL14	0.30	3.43E-04	-0.10	1.61E-03	0.02	8.56E-01	0.02	9.14E-01	0.50	5.46E-07		
METTL15	0.27	1.18E-03	0.07	8.37E-01	0.07	9.32E-02	-0.48	1.69E-17	-0.05	6.83E-01	0.35	1.39E-02
METTL16	0.14	6.24E-02	-0.64	1.48E-14	-0.09	2.78E-01	0.22	6.50E-14	-0.22	8.58E-03	-0.11	3.79E-01
METTL17	-3.56	1.67E-06	0.18	2.05E-02	-0.16	4.08E-01	-0.71	1.28E-21	0.41	1.55E-04	-0.22	8.72E-02
METTL18	0.25	3.63E-02	0.15	3.74E-02			0.20	8.41E-03	-1.03	1.16E-07		
TRMT44			0.14	2.01E-01								
METTL21A	-0.29	2.30E-01	0.63	1.53E-10					-1.33	5.48E-04		
VCPKMT			-0.02	4.23E-01							-0.36	6.80E-03
METTL25			0.20	3.80E-03					-0.19	5.49E-01		
METTL26	0.20	2.39E-01	-0.22	1.83E-02	-0.23	8.56E-08	0.72	2.31E-28	0.04	7.12E-01	-0.21	4.38E-02

Table S9. Correlation between copy number and mRNA expression of METTLs in CPTAC-LUAD tumor samples

METTL gene	Pearson Correlation	P - value	FDR (BH)	Spearman Correlation	P - value	FDR (BH)
METTL1	0.798	2.80E-25	1.81E-23	0.541	1.25E-09	5.13E-09
METTL2A	0.680	4.05E-16	3.88E-15	0.699	2.93E-17	3.28E-16
METTL2B	0.791	1.29E-24	7.10E-23	0.734	1.11E-19	1.65E-18
METTL3	0.716	2.05E-18	2.94E-17	0.674	9.37E-16	8.57E-15
METTL4	0.751	4.87E-21	1.15E-19	0.677	0.00E+00	0.00E+00
METTL5	0.525	4.72E-09	1.60E-08	0.485	9.24E-08	2.91E-07
METTL6	0.595	9.09E-12	4.41E-11	0.553	4.40E-10	1.91E-09
METTL7A	0.092	3.41E-01	4.06E-01	0.080	4.10E-01	4.80E-01
METTL7B	0.270	4.59E-03	7.81E-03	0.236	1.33E-02	2.17E-02
METTL8	0.479	1.37E-07	3.91E-07	0.440	1.73E-06	4.63E-06
METTL9	0.547	7.76E-10	2.88E-09	0.580	3.90E-11	1.95E-10
EEF1AKMT2	0.678	5.28E-16	4.95E-15	0.652	1.58E-14	1.23E-13
NTMT1	0.450	9.35E-07	2.43E-06	0.412	8.67E-06	2.15E-05
METTL11B	0.100	3.99E-01	4.65E-01	0.123	3.00E-01	3.66E-01
CSKMT	0.505	2.11E-08	6.61E-08	0.469	2.66E-07	7.92E-07
EEF1AKNMT	0.697	3.90E-17	4.49E-16	0.684	0.00E+00	0.00E+00
METTL14	0.662	4.51E-15	3.60E-14	0.633	1.59E-13	1.08E-12
METTL15	0.583	2.96E-11	1.34E-10	0.591	1.28E-11	6.83E-11
METTL16	0.641	5.90E-14	3.96E-13	0.616	0.00E+00	0.00E+00
METTL17	0.689	1.18E-16	1.25E-15	0.665	2.94E-15	2.51E-14
METTL18	0.416	7.01E-06	1.67E-05	0.405	1.28E-05	3.12E-05
TRMT44	0.596	8.13E-12	3.96E-11	0.561	2.26E-10	1.02E-09
ETFBKMT	0.390	2.71E-05	6.06E-05	0.435	2.32E-06	6.13E-06
METTL21A	0.495	4.62E-08	1.39E-07	0.461	4.57E-07	1.32E-06
EEF1AKMT3	0.626	3.39E-13	2.00E-12	0.342	2.77E-04	5.71E-04
METTL21C	0.299	1.60E-03	2.91E-03	0.356	1.62E-04	3.43E-04
VCPKMT	0.766	2.94E-22	9.36E-21	0.669	1.97E-15	1.73E-14
METTL22	0.567	1.27E-10	5.23E-10	0.564	1.68E-10	7.71E-10
METTL23	0.752	4.30E-21	1.03E-19	0.751	4.95E-21	8.28E-20
METTL24	0.319	7.10E-04	1.35E-03	0.302	1.39E-03	2.63E-03
METTL25	0.519	7.64E-09	2.52E-08	0.450	8.96E-07	2.48E-06
RRNAD1	0.635	1.21E-13	7.75E-13	0.570	0.00E+00	0.00E+00
METTL26	0.488	7.45E-08	2.19E-07	0.486	8.17E-08	2.59E-07
METTL27	0.402	1.46E-05	3.36E-05	0.501	2.99E-08	1.01E-07

Table S10. Correlation between mRNA expression and protein abundance of METTLs in CPTAC-LUAD tumor samples

METTL gene	Pearson Correlation	P-value	FDR (BH)	Spearman Correlation	P-value	FDR (BH)
METTL1	0.422	4.44E-06	7.21E-06	0.501	3.94E-08	7.86E-08
METTL2A	NA	NA	NA	NA	NA	NA
METTL2B	0.275	3.63E-03	4.75E-03	0.221	2.03E-02	2.52E-02
METTL3	0.651	1.30E-14	4.37E-14	0.646	0.00E+00	0.00E+00
METTL4	NA	NA	NA	NA	NA	NA
METTL5	0.401	3.32E-05	5.09E-05	0.462	1.18E-06	2.07E-06
METTL6	NA	NA	NA	NA	NA	NA
METTL7A	0.728	2.08E-19	1.12E-18	0.784	4.24E-24	1.46E-23
METTL7B	0.864	5.73E-34	2.23E-32	0.882	0.00E+00	0.00E+00
METTL8	NA	NA	NA	NA	NA	NA
METTL9	0.627	4.77E-12	1.26E-11	0.562	2.71E-09	5.97E-09
EEF1AKMT2	NA	NA	NA	NA	NA	NA
NTMT1	0.671	9.88E-16	3.69E-15	0.666	0.00E+00	0.00E+00
METTL11B	NA	NA	NA	NA	NA	NA
CSKMT	NA	NA	NA	NA	NA	NA
EEF1AKNMT	0.656	7.81E-15	2.68E-14	0.627	0.00E+00	0.00E+00
METTL14	0.569	9.26E-11	2.19E-10	0.556	2.77E-10	6.57E-10
METTL15	0.337	3.21E-04	4.55E-04	0.220	2.08E-02	2.57E-02
METTL16	0.757	1.02E-21	7.16E-21	0.704	9.19E-18	2.78E-17
METTL17	0.255	8.37E-03	1.07E-02	0.221	2.29E-02	2.81E-02
METTL18	0.198	3.77E-02	4.54E-02	0.258	6.72E-03	8.67E-03
TRMT44	NA	NA	NA	NA	NA	NA
ETFBKMT	NA	NA	NA	NA	NA	NA
METTL21A	0.172	1.79E-01	2.01E-01	0.166	1.94E-01	2.16E-01
EEF1AKMT3	NA	NA	NA	NA	NA	NA
METTL21C	NA	NA	NA	NA	NA	NA
VCPKMT	NA	NA	NA	NA	NA	NA
METTL22	NA	NA	NA	NA	NA	NA
METTL23	NA	NA	NA	NA	NA	NA
METTL24	NA	NA	NA	NA	NA	NA
METTL25	0.156	3.43E-01	3.72E-01	0.239	1.43E-01	1.62E-01
RRNAD1	NA	NA	NA	NA	NA	NA
METTL26	0.491	5.01E-08	9.41E-08	0.505	1.90E-08	3.90E-08
METTL27	NA	NA	NA	NA	NA	NA

Table S11. Global and individual tumor type survival Z-scores in PRECOG dataset.

METTL	Unweighted meta-Z of all cancers	Adrenocortical cancer	Bladder cancer	Brain cancer Astrocytoma	Brain cancer Glioblastoma	Brain cancer Glioma	Brain cancer Medulloblastoma	Brain cancer Meningioma	Brain cancer Neuroblastoma	Breast cancer	Colon cancer	Gastric cancer	Germ cell tumors	Head and neck cancer
METTL1	6.16	-0.33	2.79	0.87	2.62	1.96	1.17	1.37	9.45	3.78	-2.03	0.36	0.08	-0.78
METTL7B	4.91	0.2	1.08	3.55	0.29	6.25	-	-0.21	3.91	2.18	-1.31	-0.22	1.52	0.52
METTL5	4.03	0.34	-0.51	0.94	-0.92	-0.56	-	0.12	5.61	0.94	0.77	1.23	1.89	2.13
NTMT1	3.22	-0.21	0.66	3.25	-1.73	1	-	1.08	-	4.05	-1.2	0.51	-0.84	1.44
METTL2B	2.97	-	0.16	-0.28	0.02	-	-	-	3.11	3.71	0.26	-	-	-
METTL2A	2.53	-	-1.7	-	-	-	-	-	4.74	-0.06	-2.01	-	-	-
EEF1AKNMT	2.48	0.04	-0.86	-1.36	-1.95	-2.58	-	0.41	6.72	0.7	-1.06	1.35	1.39	0.25
METTL6	2.09	-1.27	-0.13	-	-1.06	-2.18	-	2.13	3.1	0.35	0.42	1.51	-	-0.52
METTL8	1.79	0.82	-1.57	-0.48	0.41	0.19	-	0.92	4.29	-0.35	-0.51	0.17	-0.92	-0.04
METTL15	1.23	-	-	-	-	-	-	-	-	0	-	-	-	-
TRMT44	0.81	-	-	-	-	-	-	-	-	0.49	-	-	-	-
METTL23	0.58	-	-	-	-	-	-	-	-	1.4	-	-	-	-
METTL22	0.56	-	-	-	-0.94	-	-	-	-	1.92	-	-	-	-
METTL27	0.52	-1.41	0.47	-	0.44	1.02	-	-0.65	1.26	0.67	1	-0.25	-	-0.71
ETFBKMT	0.42	-	-	-	-	-	-	-	-	0.55	-	-	-	-
CSKMT	0.26	1.7	0.95	-1.05	-1.82	-3.29	-	0.21	2.12	-1.25	0.9	-0.83	2.3	-0.96
METTL4	0.19	-0.49	0.39	2.21	0.59	1.14	-	-3	0.9	1.3	1.06	-1.26	-0.74	-1.71
METTL17	0.01	-	-	-	-0.86	-	-	-	-	0.68	-	-	-	-
METTL21C	-0.03	-	-	-	-	-	-	-	-	1.17	-	-	-	-
VCPKMT	-0.1	-	-	-	-0.23	-	-	-	-	-0.02	-	-	-	-
METTL21A	-0.11	-	-	-	-	-	-	-	-	-0.05	-	-	-	-
METTL16	-0.12	-	-	-	-0.74	-	-	-	-	0.9	-	-	-	-
EEF1AKMT3	-0.22	-	-	-	0.72	-	-	-	-	-1.25	-	-	-	-
METTL18	-0.67	-	-	-	-1.02	-	-	-	-	-1.16	-	-	-	-
EEF1AKMT2	-0.74	1.69	-0.3	-3.62	-0.44	-7.08	-	-1.06	-0.36	-0.98	1.81	0.26	-0.11	1.57
RRNAD1	-0.9	-	-	-	-1.25	-	-	-	-	-1.79	-	-	-	-
METTL14	-1.73	-0.17	-0.61	-0.74	1.43	-1.72	-	0.64	-	-1.23	0.31	-0.96	-2.8	-1.72
METTL9	-2	-0.34	-0.55	-0.63	-1.54	-4.72	-	1.07	1.92	-2.74	-0.39	-0.04	1.06	-0.83
METTL3	-2.07	2.22	-0.15	-1.24	-0.32	-2.71	-	-2.58	-0.04	-1.49	-0.36	-0.26	2.7	-0.48
METTL7A	-5.75	-2.59	-3.06	-0.53	0.27	-1.43	-	0.55	-1.54	-1.81	0.23	-0.12	-4.43	-1.31

METTL	Head and neck cancer Hypopharyngeal cancer	Head and neck cancer Oesophageal cancer	Head and neck cancer Oral SCC	Hematopoietic cancer AML	Hematopoietic cancer B ALL	Hematopoietic cancer Burkitt lymphoma	Hematopoietic cancer CLL	Hematopoietic cancer DLBCL	Hematopoietic cancer FL	Hematopoietic cancer Mantle cell lymphoma	Hematopoietic cancer Multiple myeloma	Kidney cancer	Liver cancer	Liver cancer Primary
METTL1	-1.47	-	1.72	-0.45	0.56	1.79	1.42	2.17	1.95	-	1.58	-	0.43	0.11
METTL7B	-	-0.84	-0.05	3.55	-2.39	-	1.59	4.65	2.16	-	0.28	-	-	-
METTL5	-	-	0.53	2.25	0.85	1.46	-0.89	2.67	-0.3	-0.23	1.72	-	-0.32	-
NTMT1	-	-	1.83	-0.99	-0.97	-	0.56	1.76	1.64	-	1.13	-	-0.57	-
METTL2B	-	1.17	-	0.01	-	-	-	-0.31	-2.29	-	2.03	-	0.18	-0.42
METTL2A	-	-	-	0.67	0.82	-	-	-	-	-	3.99	-0.04	-	-
EEF1AKNMT	-0.09	1.1	1.59	0.12	1.47	0.7	0.81	3.12	2.54	-	3.61	-	-1.15	-
METTL6	-	0.03	0.55	2.75	-0.78	-	0.88	1.98	-	-	1.08	-	-0.39	-
METTL8	-	-	0.25	1.31	0.74	1.48	-0.89	4.27	1.19	-2.39	0.75	-	-0.01	-
METTL15	-	-	-	-	-	-	-	-	-	-	-	-	-	-
TRMT44	-	-	-	-	-	-	-	-	-	-	-	-	-	-
METTL23	-	-	-	-	-	-	-	-	-	-	-	-	-	-
METTL22	-	-	-	-	-	-	-	-	-	-	-	-	-	-
METTL27	-	-	-0.67	1.64	1.33	-	0.6	0.61	-	-	0.54	-	-	-
ETFBKMT	-	-	-	-	-	-	-	-	-	-	-	-	-	-
CSKMT	-	-	1.43	0.2	0.41	-	1.34	-0.02	-1.9	-	-2.39	-	-	-
METTL4	-	-	0.58	-1.19	-0.08	-0.97	0.17	2.28	-0.68	-	2.39	-1.4	-1.29	-0.28
METTL17	-	-	-	-	-	-	-	-	-	-	-	-	-	-
METTL21C	-	-	-	-	-	-	-	-	-	-	-	-	-	-
VCPKMT	-	-	-	-	-	-	-	-	-	-	-	-	-	-
METTL21A	-	-	-	-	-	-	-	-	-	-	-	-	-	-
METTL16	-	-	-	-	-	-	-	-	-	-	-	-	-	-
EEF1AKMT3	1.26	-	-	-	-	-	-	-	-	-	-	-	-	-
METTL18	-1.68	-	-	-	-	-	-	-	-	-	-	-	-	-
EEF1AKMT2	-	-	2.03	-0.25	-0.04	-	0.86	0.7	-2.14	-	-1.81	1.09	-0.93	-0.13
RRNAD1	-	-	-	-	-	-	-	-	-	-	-	-	-	-
METTL14	-	-0.41	0.08	-1.88	-0.47	-	-0.63	0.15	-0.55	-0.75	-0.38	-	-0.04	-
METTL9	-	-	0.68	1.1	0.38	-1.4	-2.85	2.16	1.67	-	-2.54	-1.81	0.49	-
METTL3	0.75	-	-2.83	1.34	0.67	0.29	0	1.83	-0.45	-	0.2	-	0.9	-1.45
METTL7A	1.72	-	-0.74	1.65	0.8	-0.45	1.62	1.25	0.31	0.43	-2.53	-3.46	-0.3	-

METTL	Lung cancer ADENO	Lung cancer LCC	Lung cancer SCC	Lung cancer SCLC	Melanoma	Melanoma Metastasis	Mesothelioma	Ovarian cancer	Pancreatic cancer	Prostate cancer	Sarcoma Ewing sarcoma	Osteo-
METTL1	1.69	1.43	-0.14	-0.91	0.92	2.5	0.46	-2.86	1.76	0.6	1.35	0.59
METTL7B	1.3	-0.79	0.97	-0.27	1.29	0.13	-	-3.21	1.23	1.32	1.19	0.85
METTL5	4.49	0.21	1.25	-	-0.28	-0.38	-	1.03	-1.44	-0.19	0.41	0.37
NTMT1	4.4	0.28	1.34	1.1	0.77	0.85	-	-3.71	-	0.9	1.15	0.61
METTL2B	1.43	-0.22	0.35	-1.63	2.56	-	-	2.24	2.53	1.55	-	2.4
METTL2A	3.93	0.02	-1.14	-1.55	0.01	-	-	2.57	3	1.46	-	1.11
EEF1AKNMT	0.84	0.13	0.55	-1.61	-0.79	1.69	-1.69	0.06	-	-0.99	0.58	-0.13
METTL6	0.95	-1.63	1.15	-1.78	0.73	1.82	-	0.78	0.26	0.97	0.19	1.16
METTL8	1.59	-0.77	-1.27	-0.41	0.07	0.82	-	0.01	-0.58	1.71	-1.47	1.84
METTL15	1.75	-	2.41	-	1.34	-	-	-	-	-	-	2.16
TRMT44	0.86	0.68	1.81	1.12	-0.16	-	-	-	-	-	-	0.24
METTL23	0.5	-	1.09	-	0.61	-	-	-	-	-	-	-
METTL22	0.52	-0.07	1.55	0.57	-0.65	-	-	0.63	-	-	-	-0.02
METTL27	0.16	-0.8	-1.41	-	-0.81	-0.5	-	-0.16	0.33	0.59	0.52	-0.59
ETFBKMT	0.09	0.11	-	0.02	0.54	-	-	-	-	-	-	1.34
CSKMT	-1.49	1.03	0.82	0.36	1.04	2.41	-	-0.33	-	-0.25	0.02	-
METTL4	0.77	-1.09	0.97	0.63	0.42	-1.31	-	-0.32	1.93	-0.21	-0.24	-0.3
METTL17	0.72	-0.73	0.22	0.2	0.02	-	-	0.28	-	-	-	-0.45
METTL21C	-0.36	1.38	-	-1.46	0.62	-	-	-	-	-	-	-1.57
VCPKMT	0.71	1.71	-1.83	0.41	0.28	-	-	-0.34	-	-	-	-1.32
METTL21A	-1.38	0.34	0.78	-1.09	-0.88	-	-	-	-	-	-	1.6
METTL16	-0.24	-	0.08	-	0.35	-	-	0.83	-	-	-	-1.95
EEF1AKMT3	0.08	0.46	-0.12	0.05	-1.55	-	-	-0.88	-	-	-	-0.12
METTL18	-0.81	-0.41	0.15	0.48	-0.23	-	-	-1.21	-	-	-	1.68
EEF1AKMT2	0.19	1.5	0.45	-0.52	0.47	0.81	-	1.87	-	-0.32	-1.72	1.89
RRNAD1	-0.44	-1.38	0.74	-1.11	0.57	-	-	-0.12	-	-	-	-0.84
METTL14	-1.53	-0.18	1.83	-	1.21	-1.57	-	1.14	-	-0.44	-0.78	1.95
METTL9	-0.67	0.22	1.79	-1.38	-1.03	1.55	-	-2.19	-0.05	-2.59	1.28	0.42
METTL3	-1.38	0.56	-2.66	-	-1.44	-1.58	-1.08	0.34	0.68	-1.59	-2.05	0.71
METTL7A	-4.86	-0.56	-0.73	-0.8	-1	-3.2	-2.12	-0.16	-1.28	-2.15	-2.78	-0.81

Table S12. Frequency (%) of METTL genetic amplifications, deep deletions and mutations in more than 1,000 CCLE tumor lines

METTL Gene	Amplification	Deep Deletion	Mutation
METTL1	3.11	0.47	0.00
METTL2A	4.47	0.57	1.66
METTL2B	6.31	2.59	1.21
METTL3	2.43	4.34	2.04
METTL4	4.37	5.90	1.97
METTL5	1.26	0.47	0.89
METTL6	1.17	6.78	1.34
METTL7A	1.17	0.76	0.70
METTL7B	0.97	0.47	0.83
METTL8	1.46	0.47	1.97
METTL9	1.17	2.30	1.15
EEF1AKMT2	0.78	5.41	0.45
NTMT1	0.78	2.20	1.15
METTL11B	4.27	0.28	0.96
CSKMT	0.58	1.14	0.76
EEF1AKNMT	4.17	0.09	2.36
METTL14	0.49	2.79	1.08
METTL15	1.84	2.30	2.10
METTL16	0.29	11.84	3.31
METTL17	2.91	4.24	1.97
METTL18	4.08	0.19	2.17
TRMT44	0.58	6.49	3.12
ETFBKMT	6.31	1.72	1.53
METTL21A	0.29	1.24	0.96
EEF1AKMT3	3.11	0.47	0.76
METTL21C	5.05	5.89	1.15
VCPKMT	3.88	3.17	1.02
METTL22	0.97	2.00	2.36
METTL23	4.08	0.47	0.70
METTL24	0.58	7.37	1.59
METTL25	0.97	2.20	2.87
RRNAD1	4.95	0.28	1.91
METTL26	0.39	3.46	1.46
METTL27	5.15	2.10	0.64

Table S13. List of proteins with significant (FDR < 0.01) correlation with METTL1 protein expression in all three cohorts: CPTAC-LUAD, CPTAC-BRCA, and CCLE

CPTAC-LUAD				CPTAC-BRCA				CCLE			
Protein	Spearman *rho*	p-value	FDR (BH)	Protein	Spearman *rho*	p-value	FDR (BH)	Protein	Spearman *rho*	p-value	FDR (BH)
WDR4	0.814	1.00E-11	1.00E-07	WDR4	0.700	1.00E-09	1.00E-06	WDR4	0.600	0.00E+00	0.00E+00
CLUH	0.470	3.04E-07	2.10E-04	CLUH	0.431	9.14E-07	2.06E-04	CLUH (I3L2B0)	0.368	4.44E-11	2.05E-09
CLUH	0.470	3.04E-07	2.10E-04	CLUH	0.431	9.14E-07	2.06E-04	CLUH (O75153)	0.379	5.45E-14	7.55E-12
ZWILCH	0.450	1.67E-06	5.06E-04	ZWILCH	0.337	1.45E-04	4.62E-03	ZWILCH	0.304	3.15E-09	7.11E-08
KIF23	0.436	4.23E-06	8.08E-04	KIF23	0.371	2.98E-05	1.66E-03	KIF23	-0.166	1.36E-03	5.39E-03
PSME4	0.430	3.38E-06	7.74E-04	PSME4	0.415	2.53E-06	3.33E-04	PSME4	0.331	9.33E-11	3.73E-09
EIF4A1	0.427	4.19E-06	8.08E-04	EIF4A1	0.477	4.11E-08	3.45E-05	EIF4A1	0.393	3.26E-15	7.04E-13
CSE1L	0.421	5.60E-06	9.71E-04	CSE1L	0.364	4.27E-05	2.12E-03	CSE1L	0.378	7.41E-14	9.74E-12
CHORDC1	0.419	6.37E-06	1.06E-03	CHORDC1	0.316	3.88E-04	8.14E-03	CHORDC1	0.295	8.72E-09	1.70E-07
PSMG2	0.416	7.44E-06	1.18E-03	PSMG2	0.520	1.26E-09	5.27E-06	PSMG2	0.422	0.00E+00	0.00E+00
RRM2	0.412	9.28E-06	1.38E-03	RRM2	0.483	2.72E-08	2.55E-05	RRM2	0.213	3.77E-05	2.45E-04
TELO2	0.412	9.61E-06	1.38E-03	TELO2	0.323	3.02E-04	7.05E-03	TELO2	0.340	2.46E-11	1.25E-09
NAA25	0.406	1.26E-05	1.59E-03	NAA25	0.323	3.07E-04	7.10E-03	NAA25	0.427	0.00E+00	0.00E+00
NCAPD2	0.405	1.38E-05	1.61E-03	NCAPD2	0.393	8.85E-06	7.72E-04	NCAPD2	0.322	2.88E-10	9.45E-09
POP1	0.397	2.11E-05	2.20E-03	POP1	0.401	5.82E-06	5.84E-04	POP1	0.168	1.18E-03	4.79E-03
DARS	0.395	2.34E-05	2.37E-03	DARS	0.429	1.06E-06	2.12E-04	DARS	0.348	7.79E-12	4.94E-10
RPS2	0.394	2.37E-05	2.37E-03	RPS2	0.401	4.61E-06	4.84E-04	RPS2	0.409	0.00E+00	0.00E+00
GART	0.387	2.98E-05	2.65E-03	GART	0.312	5.05E-04	9.77E-03	GART	0.414	0.00E+00	0.00E+00
XPOT	0.385	3.77E-05	3.09E-03	XPOT	0.380	1.88E-05	1.21E-03	XPOT	0.419	0.00E+00	0.00E+00
NCAPG	0.380	4.87E-05	3.42E-03	NCAPG	0.380	1.82E-05	1.20E-03	NCAPG	0.326	1.72E-10	6.05E-09
XPO5	0.376	5.73E-05	3.73E-03	XPO5	0.451	2.53E-07	9.65E-05	XPO5	0.350	5.92E-12	3.99E-10
SMC2	0.376	5.92E-05	3.73E-03	SMC2	0.336	1.69E-04	5.08E-03	SMC2	0.224	1.54E-05	1.12E-04
SHMT2	0.368	8.54E-05	4.73E-03	SHMT2	0.472	6.15E-08	4.29E-05	SHMT2	-0.159	2.17E-03	8.04E-03
NSUN2	0.367	9.07E-05	4.85E-03	NSUN2	0.427	1.21E-06	2.30E-04	NSUN2	0.345	1.24E-11	7.38E-10
RPL5	0.365	9.68E-05	5.07E-03	RPL5	0.347	9.73E-05	3.64E-03	RPL5	0.365	6.29E-13	6.12E-11
MRPL45	0.365	9.88E-05	5.12E-03	MRPL45	0.322	3.19E-04	7.23E-03	MRPL45	-0.205	7.59E-05	4.51E-04
NCAPH	0.365	1.00E-04	5.17E-03	NCAPH	0.321	3.39E-04	7.51E-03	NCAPH	0.170	1.04E-03	4.28E-03
CAD	0.362	1.15E-04	5.58E-03	CAD	0.319	3.66E-04	7.86E-03	CAD	0.396	1.37E-15	3.17E-13
ABCF2	0.360	1.14E-04	5.57E-03	ABCF2	0.460	1.40E-07	7.23E-05	ABCF2	0.352	4.27E-12	3.01E-10
TRMT6	0.356	1.47E-04	6.38E-03	TRMT6	0.368	3.54E-05	1.88E-03	TRMT6	0.205	7.64E-05	4.53E-04
SMC4	0.355	1.39E-04	6.19E-03	SMC4	0.357	6.00E-05	2.63E-03	SMC4	0.252	1.03E-06	1.07E-05
MKI67	0.354	1.59E-04	6.62E-03	MKI67	0.351	8.36E-05	3.28E-03	MKI67	-0.209	5.36E-05	3.35E-04
CMSS1	0.346	2.35E-04	8.25E-03	CMSS1	0.355	6.58E-05	2.81E-03	CMSS1	0.230	8.87E-06	6.93E-05
SLIRP	0.344	2.49E-04	8.50E-03	SLIRP	0.324	2.68E-04	6.57E-03	SLIRP	-0.178	6.01E-04	2.67E-03
PSMG1	0.343	2.62E-04	8.80E-03	PSMG1	0.484	2.51E-08	2.55E-05	PSMG1	0.411	0.00E+00	0.00E+00
HSP90AB1	0.343	2.63E-04	8.81E-03	HSP90AB1	0.357	5.95E-05	2.63E-03	HSP90AB1	0.357	2.27E-12	1.75E-10
TRIP13	0.341	2.86E-04	9.14E-03	TRIP13	0.427	1.17E-06	2.30E-04	TRIP13	0.337	4.24E-11	1.98E-09
PUS1	0.340	3.08E-04	9.56E-03	PUS1	0.332	2.04E-04	5.65E-03	PUS1	0.193	2.04E-04	1.05E-03
NAA15	0.337	3.21E-04	9.80E-03	NAA15	0.322	3.15E-04	7.19E-03	NAA15	0.474	0.00E+00	0.00E+00
HMGN4	-0.463	4.76E-07	2.73E-04	HMGN4	-0.467	8.77E-08	5.26E-05	HMGN4	-0.156	2.63E-03	9.52E-03

Supplementary Figure Legends:

Figure S1. Phylogenetic Tree of 34 METTL proteins. The multiple sequence alignment was performed using Clustal Omega of Multiple Sequence Alignment at website: https://www.ebi.ac.uk.

Figure S2. Boxplots showing (A) mRNA in four METTLs (METTL2B, METTL3, METTL24, and METTL26), and (B) protein difference in three METTLs (METTL2B, METTL3, and METTL26) of CPTAC-LUAD tumor and NAT samples. Blue: normal adjacent tissues (NAT); red: LUAD tumor samples. Sample numbers are indicated.

Figure S3. Correlations between (A) DNA copy number and mRNA expression, as well as (B) mRNA expression and protein abundance in CPTAC-LUAD cohort.

Figure S4. Kaplan-Meier progression-free survival curve for METTL26 mRNA expression in LUAD patients.

Figure S5. Expression levels of METTL1, measured by the qRT-PCR assay, in a panel of three breast cancer cell lines (MCF7, SUM52, and SUM159), three lung cancer lines (A549, NCI-H23, and NCI-H522), and MCF10A line. mRNA expression levels in the immortalized but nontumorigenic breast epithelial cell line MCF10A cells were arbitrarily set as 1.

Figure S6. Relative protein abundance of METTL7B and NTMT1 in more than 300 CCLE lines across 22 lineages.

Figure S7. Correlation between METTL1 and WDR4 protein abundance in CPTAC-LUAD cohort.

Figure S8. Druggability of SAM binding pockets of human METTL1 (PDB: 3CKK) was estimated by DoGSiteScorer tool of the ProteinsPlus server.

Figure S9: Sequence alignment of enzymatic domain of four proteins derived from genome sequences of the following: human METTL1, Yeast Trm8, *E. coli* TrmB, and *Bacillus subtilis*

TrmB. The secondary structure of human METTL1 is shown above. Residues with 100% conservation are indicated in solid red boxes and those with identity of 70% or higher are depicted in light red color.

Figure S1

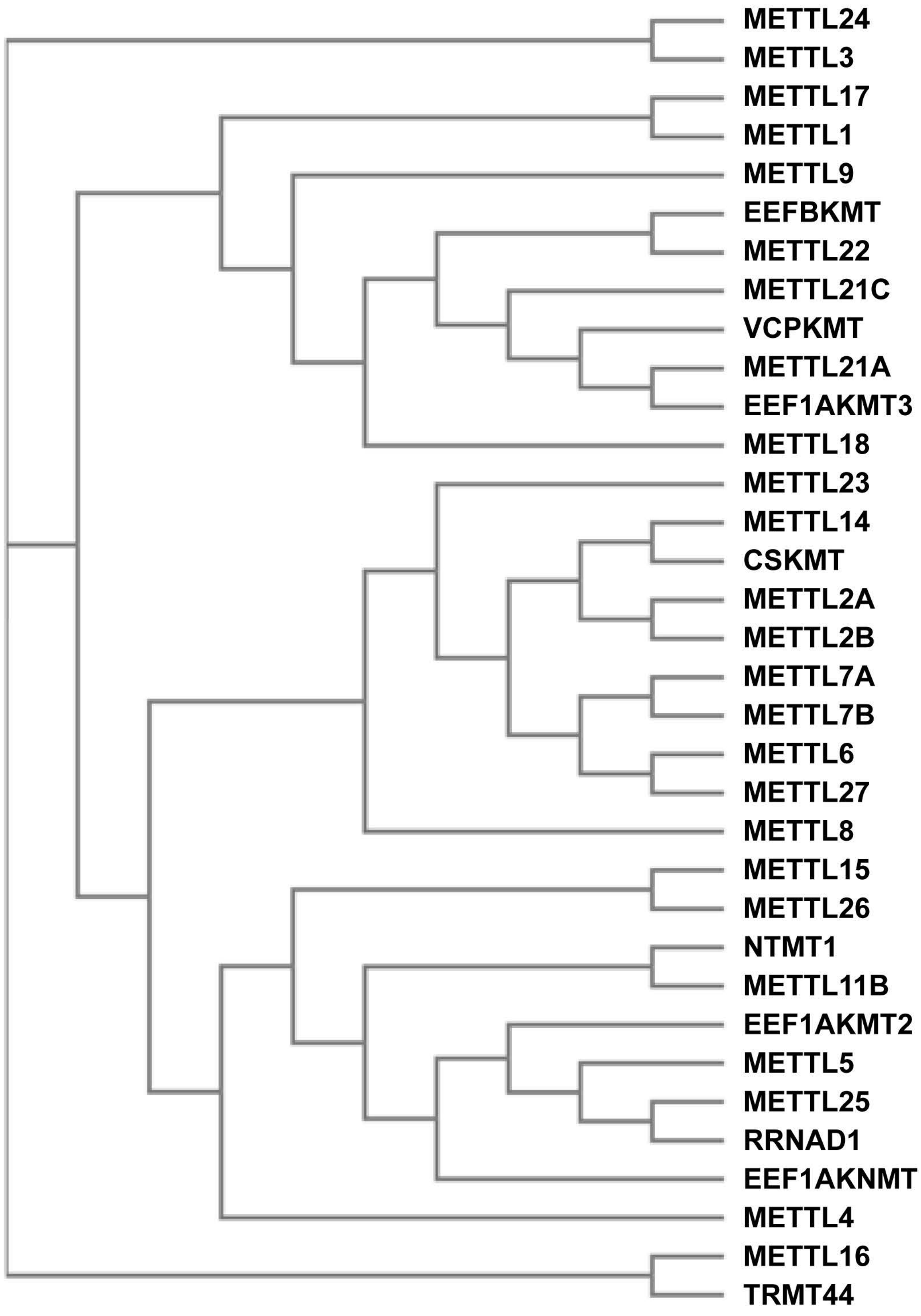
METTL24
METTL3
METTL17
METTL1
METTL9
EEFBKMT
METTL22
METTL21C
VCPKMT
METTL21A
EEF1AKMT3
METTL18
METTL23
METTL14
CSKMT
METTL2A
METTL2B
METTL7A
METTL7B
METTL6
METTL27
METTL8
METTL15
METTL26
NTMT1
METTL11B
EEF1AKMT2
METTL5
METTL25
RRNAD1
EEF1AKNMT
METTL4
METTL16
TRMT44

Figure S2

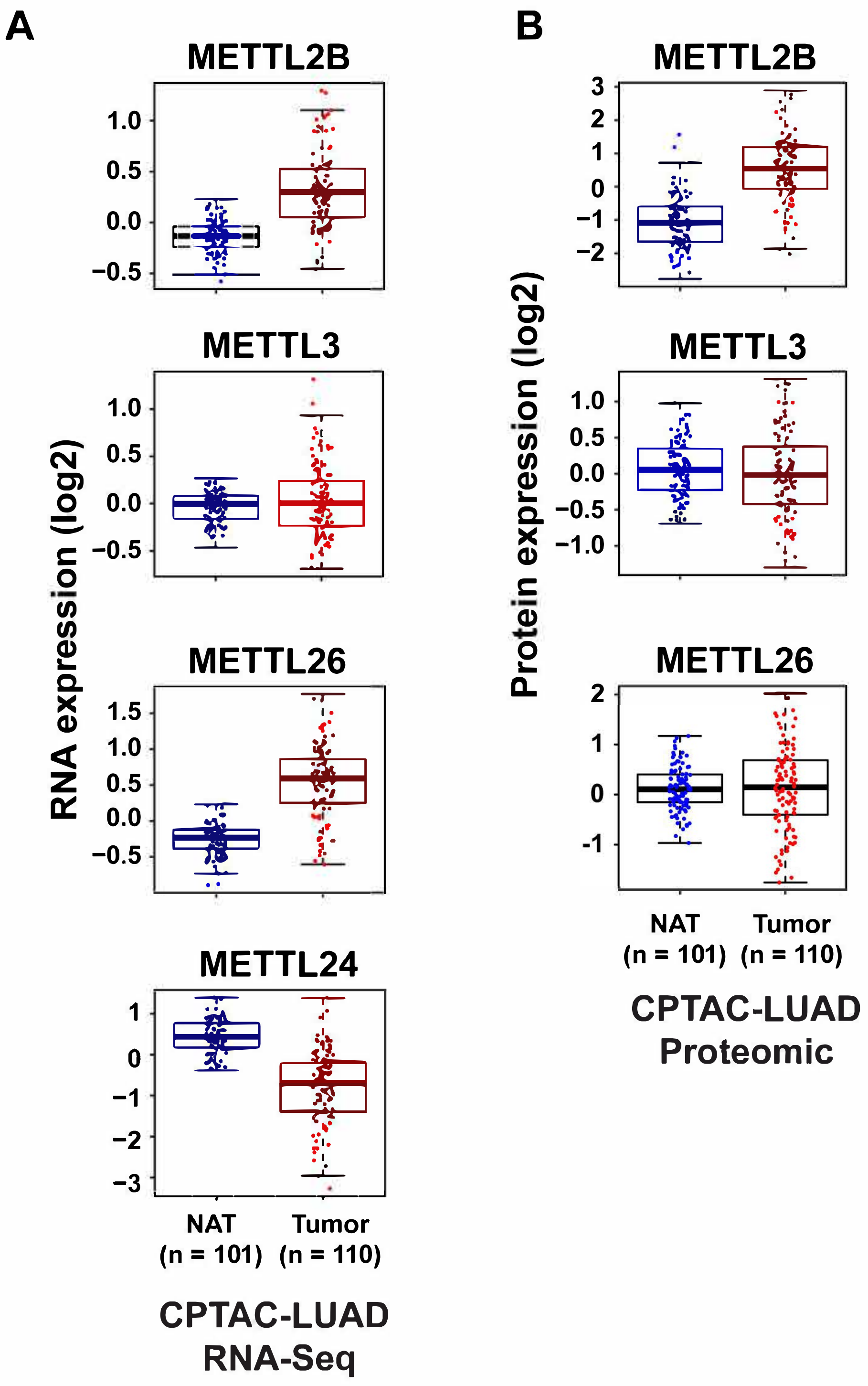
A
B
METTL2B
METTL3
METTL26
METTL24
RNA expression (log2)
Protein expression (log2)
1.0
0.5
0.0
−0.5
1.5
1
0
−1
−2
−3
3
2
−1.0
NAT
(n = 101)
Tumor
(n = 110)
CPTAC-LUAD
RNA-Seq
CPTAC-LUAD
Proteomic

Figure S3

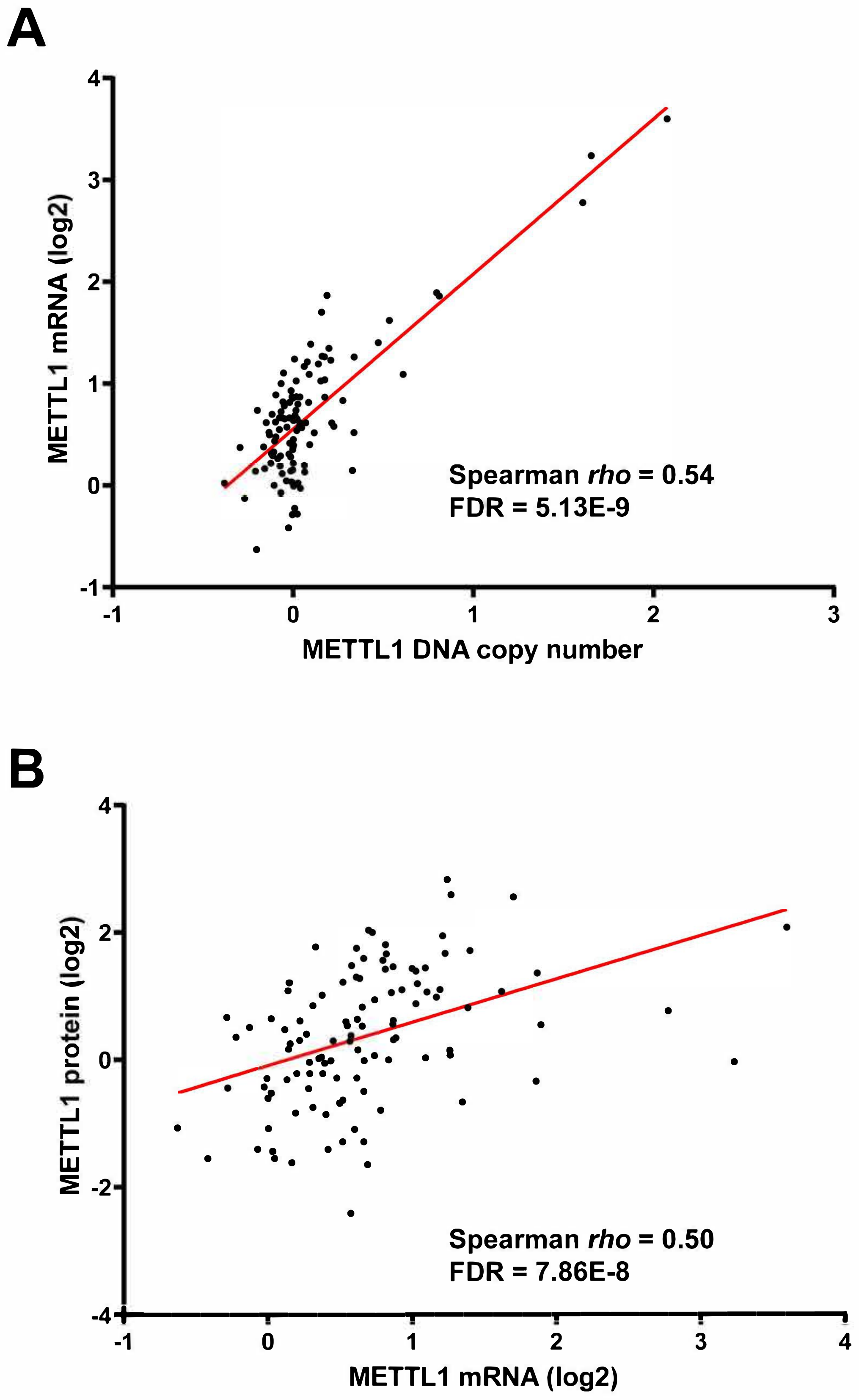
A
METTL1 mRNA (log2)
METTL1 DNA copy number
Spearman *rho* = 0.54
FDR = 5.13E-9
B
METTL1 protein (log2)
METTL1 mRNA (log2)
Spearman *rho* = 0.50
FDR = 7.86E-8

Figure S4

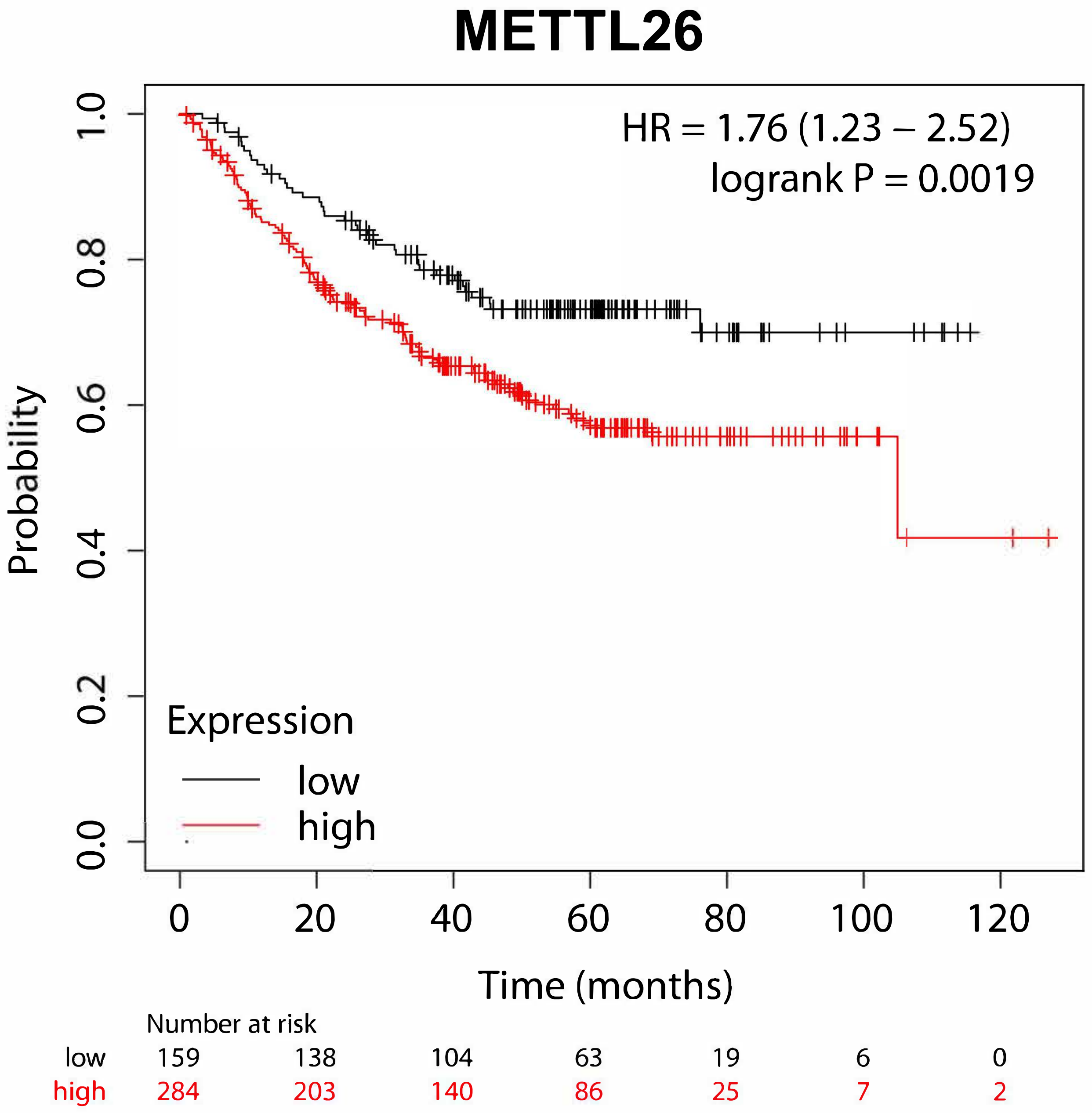
METTL26
HR = 1.76 (1.23 – 2.52)
logrank P = 0.0019
Probability
0.0
0.2
0.4
0.6
0.8
1.0
Expression
low
high
0
20
40
60
80
100
120
Time (months)
Number at risk
low 159 138 104 63 19 6 0
high 284 203 140 86 25 7 2

Figure S5

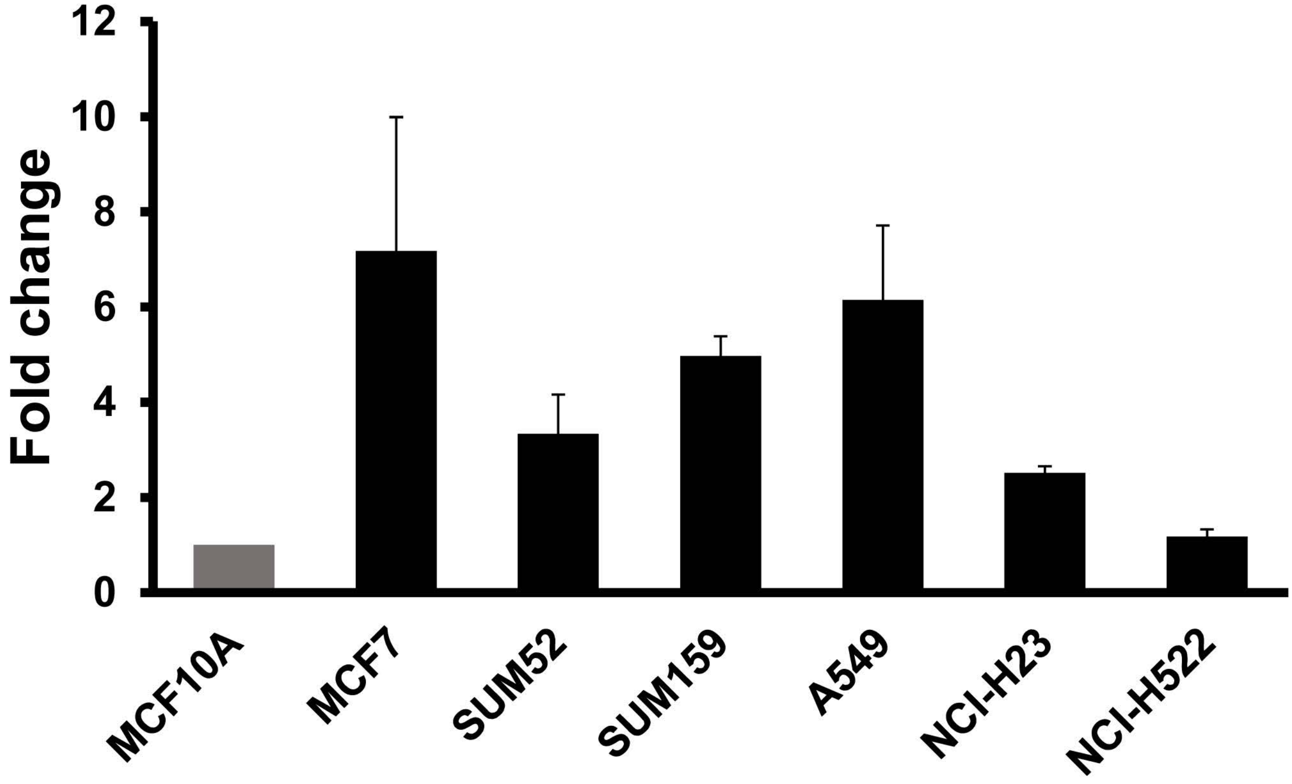

12
10
8
6
4
2
0
Fold change
MCF10A
MCF7
SUM52
SUM159
A549
NCI-H23
NCI-H522

Figure S6

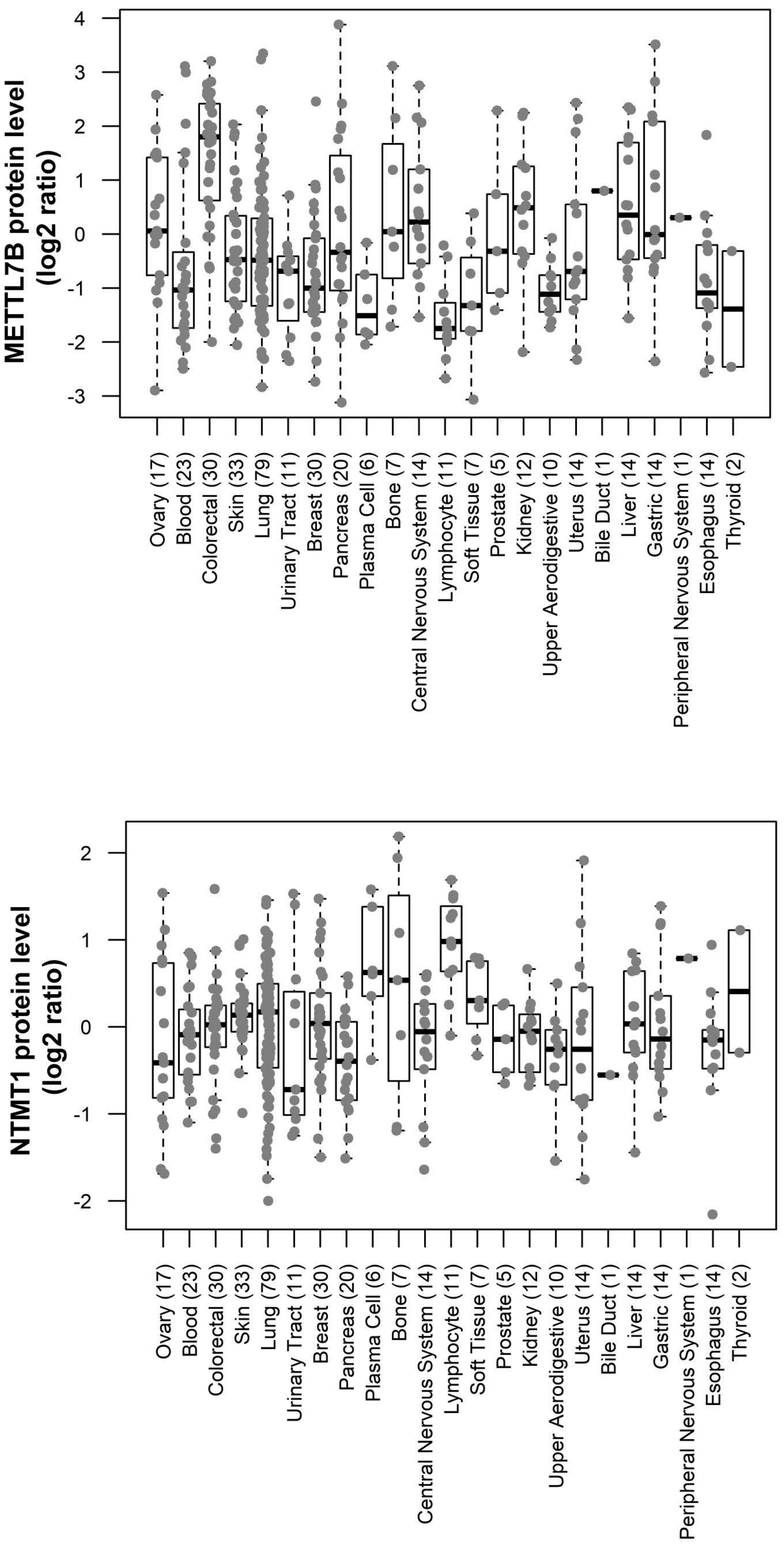
METTL7B protein level (log2 ratio)
4
3
2
1
0
-1
-2
-3
Ovary (17)
Blood (23)
Colorectal (30)
Skin (33)
Lung (79)
Urinary Tract (11)
Breast (30)
Pancreas (20)
Plasma Cell (6)
Bone (7)
Central Nervous System (14)
Lymphocyte (11)
Soft Tissue (7)
Prostate (5)
Kidney (12)
Upper Aerodigestive (10)
Uterus (14)
Bile Duct (1)
Liver (14)
Gastric (14)
Peripheral Nervous System (1)
Esophagus (14)
Thyroid (2)
NTMT1 protein level (log2 ratio)
2
1
0
-1
-2
Ovary (17)
Blood (23)
Colorectal (30)
Skin (33)
Lung (79)
Urinary Tract (11)
Breast (30)
Pancreas (20)
Plasma Cell (6)
Bone (7)
Central Nervous System (14)
Lymphocyte (11)
Soft Tissue (7)
Prostate (5)
Kidney (12)
Upper Aerodigestive (10)
Uterus (14)
Bile Duct (1)
Liver (14)
Gastric (14)
Peripheral Nervous System (1)
Esophagus (14)
Thyroid (2)

Figure S7

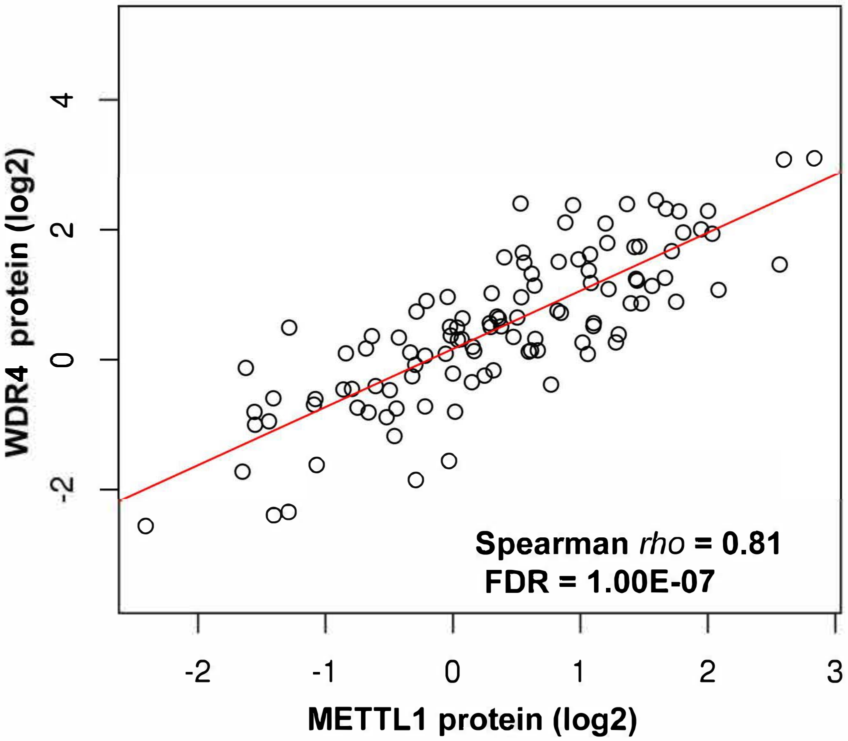

WDR4 protein (log2)
4
2
0
-2
Spearman *rho* = 0.81
FDR = 1.00E-07
-2
-1
0
1
2
3
METTL1 protein (log2)

Figure S8

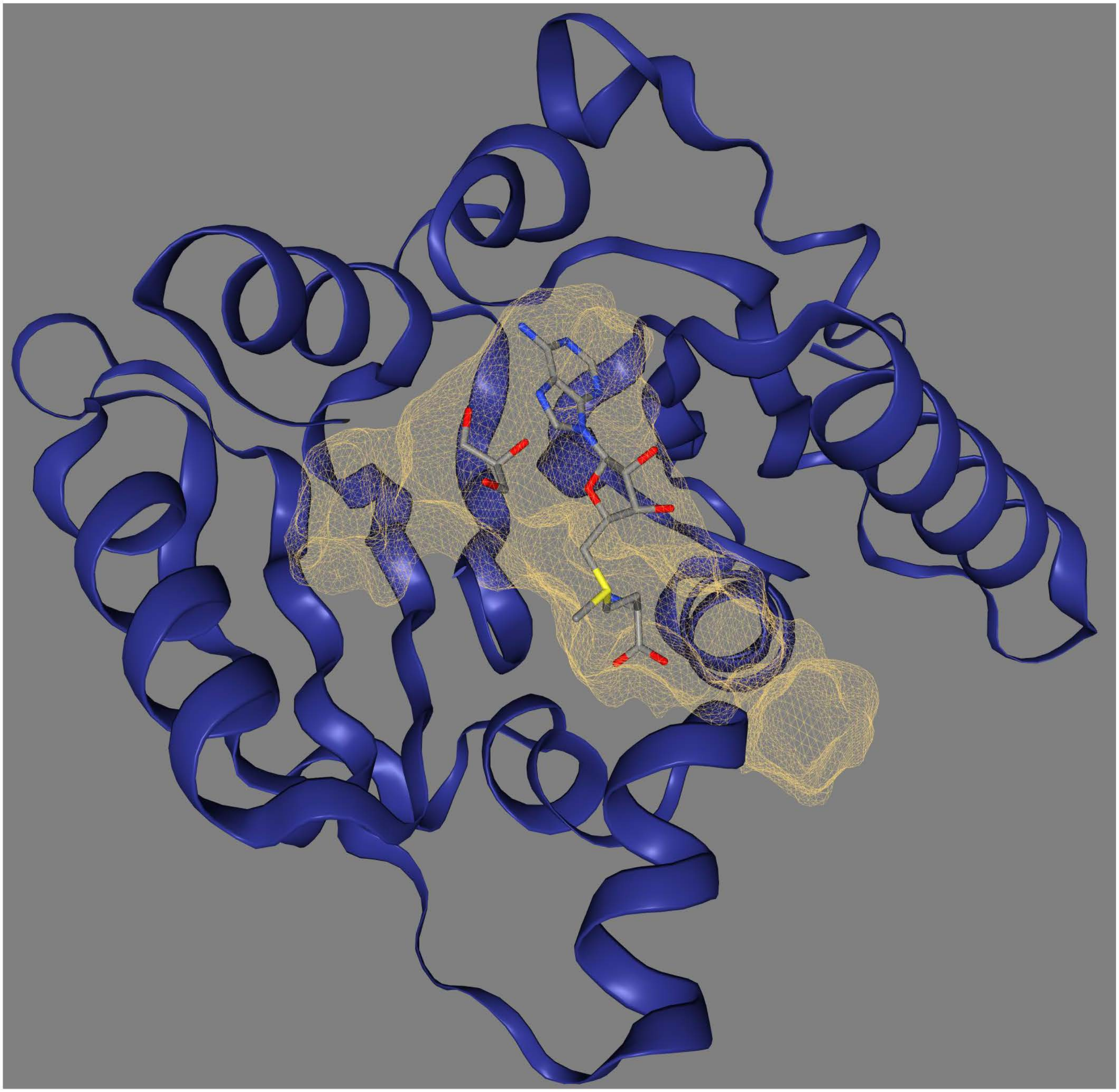

Figure S9

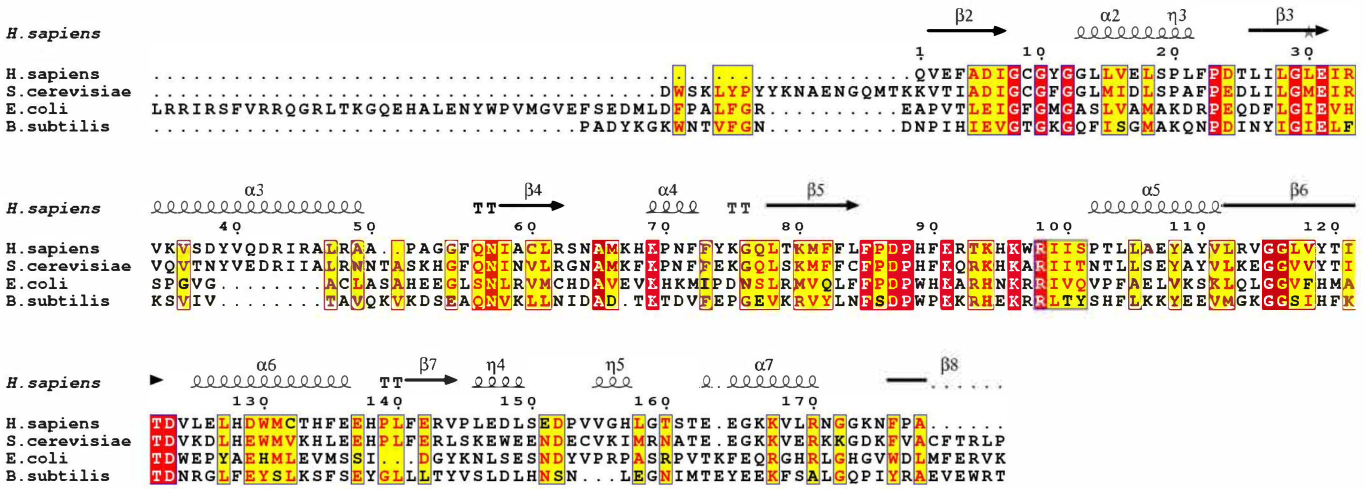
H.sapiens
S.cerevisiae
E.coli
B.subtilis
β2
α2
η3
β3
α3
β4
α4
β5
α5
β6
α6
β7
η4
η5
α7
β8
